# Efficient production of antifungal proteins in plants using a new transient expression vector derived from tobacco mosaic virus

**DOI:** 10.1111/pbi.13038

**Published:** 2018-12-06

**Authors:** Xiaoqing Shi, Teresa Cordero, Sandra Garrigues, Jose F. Marcos, José‐Antonio Daròs, María Coca

**Affiliations:** ^1^ Centre for Research in Agricultural Genomics (CRAG CSIC‐IRTA‐UAB‐UB) Cerdanyola del Vallès Spain; ^2^ Instituto de Biología Molecular y Celular de Plantas (IBMCP CSIC‐Universitat Politècnica de València) Valencia Spain; ^3^ Instituto de Agroquímica y Tecnología de Alimentos (IATA, CSIC) Paterna Spain

**Keywords:** antifungal proteins, gibson assembly, *Nicotiana benthamiana*, plant biofactory, tobacco mosaic virus, viral vector

## Abstract

Fungi that infect plants, animals or humans pose a serious threat to human health and food security. Antifungal proteins (AFPs) secreted by filamentous fungi are promising biomolecules that could be used to develop new antifungal therapies in medicine and agriculture. They are small highly stable proteins with specific potent activity against fungal pathogens. However, their exploitation requires efficient, sustainable and safe production systems. Here, we report the development of an easy‐to‐use, open access viral vector based on *Tobacco mosaic virus* (TMV). This new system allows the fast and efficient assembly of the open reading frames of interest in small intermediate entry plasmids using the Gibson reaction. The manipulated TMV fragments are then transferred to the infectious clone by a second Gibson assembly reaction. Recombinant proteins are produced by agroinoculating plant leaves with the resulting infectious clones. Using this simple viral vector, we have efficiently produced two different AFPs in *Nicotiana benthamiana* leaves, namely the *Aspergillus giganteus* AFP and the *Penicillium digitatum* AfpB. We obtained high protein yields by targeting these bioactive small proteins to the apoplastic space of plant cells. However, when AFPs were targeted to intracellular compartments, we observed toxic effects in the host plants and undetectable levels of protein. We also demonstrate that this production system renders AFPs fully active against target pathogens, and that crude plant extracellular fluids containing the AfpB can protect tomato plants from *Botrytis cinerea* infection, thus supporting the idea that plants are suitable biofactories to bring these antifungal proteins to the market.

## Introduction

Disease‐causing fungi that infect plants, animals and humans pose a serious threat to human and animal health, food security and ecosystem resilience (Fisher *et al*., 2016, 2012). More people die every year from fungal infections than from malaria (Bongomin *et al*., 2017). Fungal infections can have fatal consequences for at‐risk immunocompromised patients with HIV/AIDS, anti‐cancer chemotherapies, corticosteroid therapies, and organ transplantation, among others (Campoy and Adrio, 2017). In addition, fungi are a challenge to food security because they destroy major crops globally and contaminate food and feed with mycotoxins that are detrimental to animal and human health (Bebber and Gurr, 2015). Only a few classes of antifungal agents are available today, and even these are not fully effective due to the development of resistance, host toxicity, and undesirable side effects (Perfect, 2016). There is thus an urgent need to develop novel antifungals, whose properties and mechanisms of action represent improvements on the existing ones, and which can be applied in diverse fields, including crop and postharvest protection, preservation in cosmetics, materials and food, and animal and human health.

Antifungal proteins (AFPs) produced by filamentous fungi are a specific class of antimicrobial peptides (AMPs). Antifungal proteins are promising biomolecules that could be used to develop new antifungal therapies in medicine and agriculture (Garrigues *et al*., 2017; Huber *et al*., 2018; López‐García *et al*., 2012; Meyer, 2008). AFPs are small proteins, usually cationic, that are rich in cysteine residues, and are folded in compact structures supported by disulphide bridges, which make them highly stable and resistant to heat, proteases and extreme pH (Batta *et al*., 2009; Campos‐Olivas *et al*., 1995; Garrigues *et al*., 2017; Hegedüs and Marx, 2013). They exhibit potent specific antifungal activity at very low concentrations against important human and plant fungal pathogens (Garrigues *et al*., 2017; Huber *et al*., 2018; Marx *et al*., 2008; Tóth *et al*., 2016; Vila *et al*., 2001; Virágh *et al*., 2014), and do not have toxic effects on plant or mammalian cells (Moreno *et al*., 2006; Szappanos *et al*., 2006). However, the exploitation of AFPs requires efficient, sustainable and safe production systems. Antifungal proteins have been biotechnologically produced in *Pichia pastori* at relatively low yield, and more efficiently in filamentous fungi using a *Penicillium chrysogenum*‐based expression cassette (Garrigues *et al*., 2018; Huber *et al*., 2018; López‐García *et al*., 2010; Sonderegger *et al*., 2016; Tóth *et al*., 2018; Virágh *et al*., 2014). Plants represent also a good option for producing AFPs that are cysteine‐rich proteins that require disulphide bridges formation and proper folding for activity. Moreover, previous reports indicate that plants can sustain AFP production (Coca *et al*., 2004; Girgi *et al*., 2006; Moreno *et al*., 2005; Oldach *et al*., 2001). These reports show that AFPs can be heterologously produced in plants, leading to improve resistance to fungal pathogens.

Plants are excellent biofactories for producing proteins and other metabolites of interest for research, pharma and industry. They are fueled by sunlight, are free from human pathogens, and compared to other systems, production can be scaled up easily. Many biotechnological tools have been developed for molecular farming, and those derived from plant viruses are prominent among them (Hefferon, 2017). Higher plants host a remarkable diversity of viruses with different genomes and strategies for genome replication and expression. However, they all have small genomes, and thus have limited capacity to harbor genetic information. Despite this limitation, plant viruses can complete complex and tightly regulated infectious processes in their host plants. Definitively, evolution has favored small and simple, but still powerful, genetic elements in plant virus genomes, a wealthy source of parts for plant biotechnology and synthetic biology.

A pioneering example of a plant virus transformed into a biotechnological tool is *Tobacco mosaic virus* (TMV). This is a plus strand RNA virus of genus *Tobamovirus,* within the family *Virgaviridae* (Ishibashi and Ishikawa, 2016; Knapp and Lewandowski, 2001; Scholthof, 2004). The genomic RNA is encapsidated by 2130 units of the viral coat protein (CP) into rigid, helical rod‐shape virions of about 18 nm diameter, and 300‐310 nm length. TMV RNA encodes four proteins: two 5′‐end proximal overlapping 126 and 183 kDa replication proteins that are alternatively produced through a ribosomal readthrough mechanism, the 30 kDa movement protein (MP), and the 3′‐end proximal 17.5 kDa CP. The two replication proteins are expressed from the genomic RNA, while the MP and CP are expressed from 3′ co‐terminal subgenomic RNAs. The TMV genome also contains 5′‐ and 3′‐untranslated regions that are important for virus translation and replication (Chujo *et al*., 2015).

A key property of TMV is the rapid accumulation of large amounts of the viral CP in infected plant tissues. A combination of knowledge and a trial‐and‐error approach led to the construction of TMV‐derived vectors (Dawson, 2014) such as TB‐2 (Donson *et al*., 1991; Kearney *et al*., 1993), or 30B (Shivprasad *et al*., 1999), which allow to express a foreign open reading frame (ORF) under the control of the viral CP promoter; in these cases, encapsidation is achieved by inserting into the recombinant virus an ORF encoding the CP of a different tobamovirus. Other vectors such as the TMV RNA‐based overexpression (TRBO) vector, in which most of the viral CP is replaced by the ORF of interest, produce large amounts of recombinant proteins but are incapable of spreading systemically (Lindbo, 2007). Improvements in TMV‐based vectors have also focused on increasing the efficiency of establishing the infection foci and of cloning the genes of interest. One of the most popular systems uses DNA modules delivered by *Agrobacterium tumefaciens* and assembled *in vivo* by a site‐specific recombinase to produce the TMV‐derived vector (Marillonnet *et al*., 2004). This de‐constructed system was further improved by introducing silent nucleotide substitutions and multiple introns into the TMV coding sequences, in order to increase infectivity (Marillonnet *et al*., 2005). Another improved series of TMV‐derived vectors uses Gateway cloning to facilitate insertion of the recombinant ORFs, which can also be fused to a broad series of epitope tags and fluorescent proteins (Kagale *et al*., 2012).

Here, we report the development of a new TMV‐derived vector system that allows quick and easy cloning of the ORFs of interest using the Gibson assembly reaction (Gibson *et al*., 2009). We describe how we have used this system to produce large amounts of AFPs in *Nicotiana benthamiana* plants. We show that it is important to target these bioactive proteins to the extracellular space, since their toxic effects prevent them from accumulating in intracellular compartments. Moreover, by accumulating the AFPs in the extracellular fluids, downstream purification is simpler. We show also that the recombinant AFPs produced by the new system have exactly the same activity as their native counterparts of fungal origin. Finally, we demonstrate that plant extracellular fluids containing the *Penicillium digitatum* AfpB can protect tomato plants from the grey mold disease caused by *Botrytis cinerea*.

## Results

### A new TMV‐based vector system in which ORFs of interest are inserted using the Gibson assembly reaction

**Figure 1:**
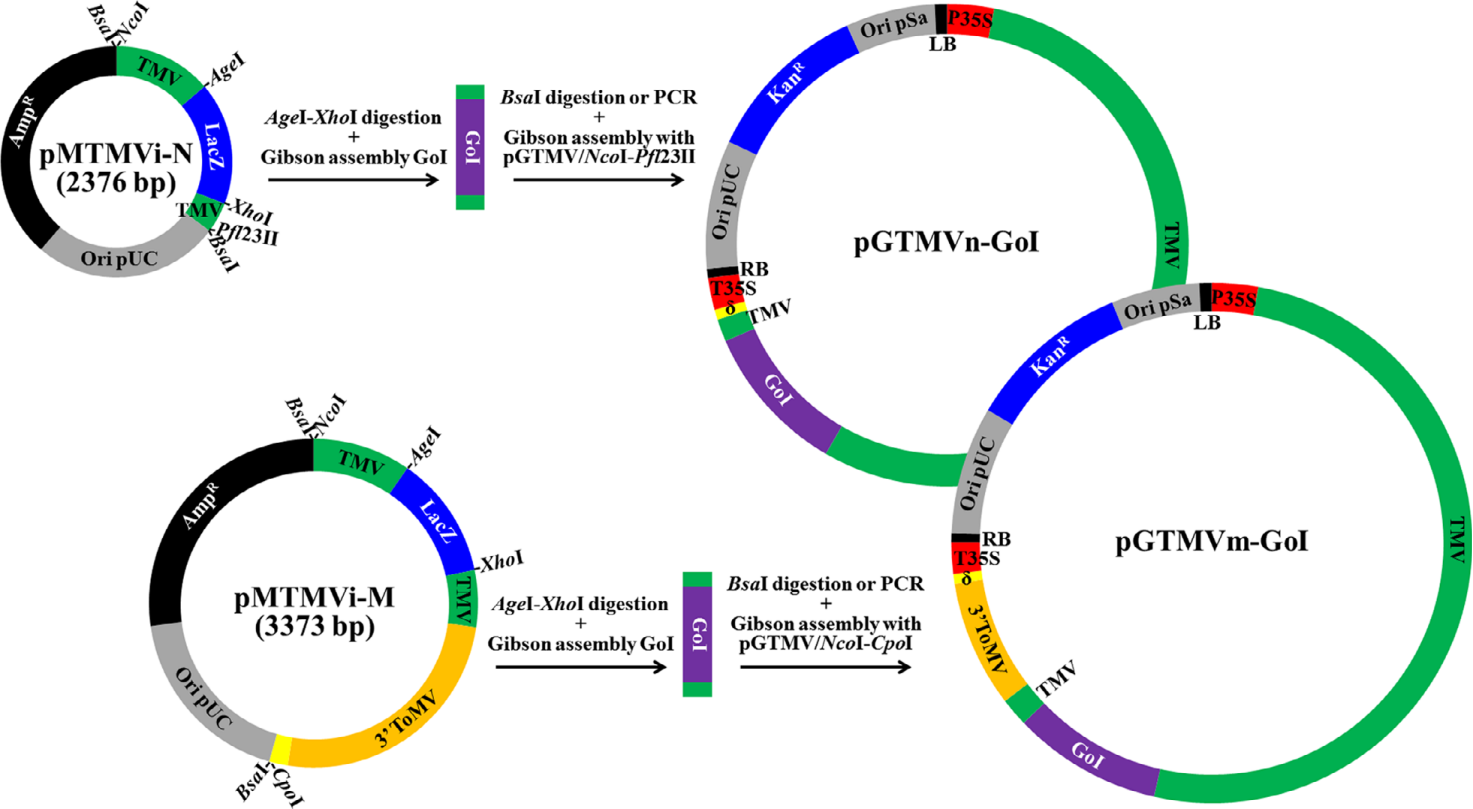
The TMV vector system, which allows Gibson assembly of the ORF of interest. Schematic representation of intermediate entry plasmids pMTMVi‐N and pMTMVi‐M, and the Gibson assembly reactions that allow insertion of the gene of interest (GoI) into pGTMV to produce the binary plasmids that will express the recombinant viruses (pGTMVn‐GoI and pGTMVm‐GoI). Ori pUC and Ori pSa, replication origins; Amp^R^ and Kan^R^, ampicillin and kanamycin resistance selection markers; LacZ, fragment of *E. coli* b‐galactosidase, which allows blue–white screening in the presence of X‐gal; TMV and 3′ ToMV, viral cDNAs; LB and RB, left and right borders of the *Agrobacterium. tumefaciens* transfer DNA; P35S and T35S, *Cauliflower mosaic virus* (CaMV) 35S promoter and terminator; δ, ribozyme.

As a first step toward a new TMV‐derived vector system for plant biotechnology that uses the Gibson assembly reaction to insert the ORFs of interest, we constructed a TMV infectious clone that efficiently infects plants when delivered by agroinoculation (pGTMV; Figures S1 and S2; Addgene plasmid # 118755). We then transferred a fragment of the TMV cDNA containing the entire CP ORF (from A5431 to T6278) to a minimal cloning vector. In the resulting plasmid, the TMV cDNA was flanked by recognition sites for the type‐IIS restriction enzyme *Bsa*I. The plasmid was manipulated to mutagenize the ATG initiation codon of the TMV CP into AGA (positions 5712‐5714), and to replace most of the CP ORF (from T5757 to T6176) by a linker consisting of the recognition sites for two unique restriction enzymes (*Age*I and *Xho*I) and the LacZ blue‐white selection marker. The resulting minimal intermediate plasmid (pMTMVi‐N; Addgene plasmid # 118756) is represented in Figure 1 (upper part) and its exact sequence in Figure S2. This small plasmid (2376 bp) allows us to easily and efficiently insert the ORFs of interest using the Gibson assembly reaction (Gibson *et al*., 2009). The option of assembling two or more DNA fragments in a single reaction allows us to fuse any desired peptide tag or protein moiety to the recombinant protein of interest. Once the cDNA for the recombinant protein is inserted into pMTMVi‐N, the manipulated TMV genome fragment can be transferred into the TMV infectious clone (pGTMV) using a second Gibson assembly reaction (Figure 1, upper part). This requires that pGTMV be digested with restriction enzymes *Nco*I and *Pfl*23II, which have unique recognition sites in this plasmid. There are two options for generating the insert from the pMTMVi‐N derivatives, the easiest being to digest the plasmid with *Bsa*I. However, if the recombinant cDNA contains *Bsa*I sites, the manipulated TMV DNA fragment can be produced by polymerase chain reaction (PCR) using primers PI and PII (Table S1), which flank the recognition sites of *Nco*I and *Pfl*23II.

In pMTMVi‐N derivatives, most of the TMV CP is replaced by the ORF for the recombinant protein, such that the recombinant viruses will be unable to move systemically in infected plants. To obtain recombinant TMV clones that can move within the plant, we constructed a new minimal intermediate plasmid by transferring the fragment in the pGTMV from A5431 in TMV cDNA to G62 in the delta ribozyme cDNA. We again mutagenized the TMV CP ATG to AGA and replaced most of the TMV CP with the *Age*I‐LacZ‐*Xho*I linker, as above. In this case, we replaced the final 30 nt of the TMV genome (from A6366 to the end) with the 3′ end of a *Tomato mosaic virus* (ToMV) infectious clone (from G5537 to the end). This fragment includes the ToMV CP promoter, CP ORF and 3′ UTR, and provides an alternative tobamovirus CP to encapsidate the recombinant virus, which allows it to move systemically (Shivprasad *et al*., 1999). This second minimal TMV intermediate plasmid, called pMTMVi‐M (Addgene plasmid # 118757), is shown in Figure 1 (lower part, full sequence in Figure S2). In pMTMVi‐M, the M indicates movement, whereas the N in pMTMVi‐N indicates nonmovement. The cDNA corresponding to the recombinant protein can be inserted into the *Age*I‐*Xho*I digested plasmid using the Gibson assembly reaction, as previously described. The manipulated TMV fragment can also be reintegrated into pGTMV, using the Gibson assembly reaction with a *Bsa*I‐digested insert, although in this case the destination plasmid (pGTMV) must be digested with *Nco*I and *Cpo*I. If the cDNA corresponding to the recombinant protein contains *Bsa*I sites, the insert can also be produced by PCR using primers PI and PIII (Table S1), which flank the *Nco*I and *Cpo*I recognition sites.

We confirmed that the new TMV vector system performed correctly by cloning the cDNA of reporter green fluorescent protein (GFP) into both the pMTMVi‐N and pMTMVi‐M intermediate plasmids, transferring the manipulated TMV fragments to pGTMV, and infiltrating *N. benthamiana* plants (Figure S3, sequences in Figure S4).

### Production of the antifungal protein AfpB in leaf apoplasts of *N. benthamiana* plants

**Figure 2:**
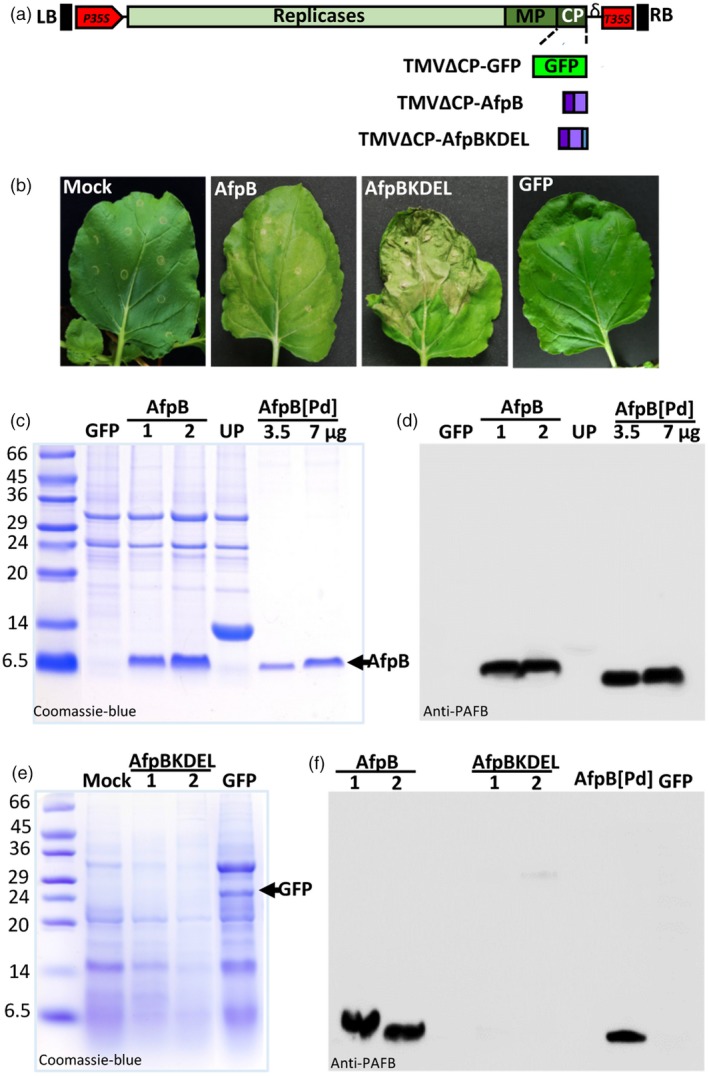
Production of AfpB in the apoplast of *Nicotiana benthamiana* leaves. (a) Schematic representation of the two constructs prepared and designed to accumulate AfpB in the apoplast or endoplasmic reticulum (ER) of plant cells. Both incorporate the signal peptide of tobacco AP24 protein at the N‐terminus of the mature AfpB protein, and the one designed for ER accumulation also contains the ER‐retention signal KDEL at its C‐terminus. RB and LB, right and left border T‐DNA; P35S and T35S, CaMV 35S promoter and terminator; δ, ribozyme; MP, movement protein; CP, coat protein. (b) Appearance of *N. benthamiana* leaves 7 days after agroinfiltration with the indicated constructs or with the agrobacterium induction media (mock). (c‐f) Analysis of AfpB accumulation in agroinfiltrated leaves from two independent plants (1 and 2). Proteins from extracellular fluids (c, d) or acid extraction (e, f) were separated by tricine‐SDS‐PAGE and Coomassie‐blue stained (c, e) or immunodetected using antibodies anti‐PAFB (d, f). Purified AfpB (3.5 or 7 μg) from *Penicillium digitatum* cultures (AfpB[Pd]) was run in parallel as a control. ECFs from plants accumulating a cysteine‐rich protein of unknown function (UP, see Figure 3) was also run in parallel for comparative purposes. Molecular weight markers are shown in kDa on the left.

AfpB is an AFP whose gene was identified in the genome of the postharvest phytopathogenic fungus *P. digitatum* but that, unlike other AFPs, is not naturally produced by the fungus (Garrigues *et al*., 2016). In a recent study, *P. digitatum* was genetically modified to produce AfpB and was found to be a highly active antifungal protein, with inhibitory concentrations up to one order of magnitude lower than other AFPs (Garrigues *et al*., 2017). Therefore, there is a great interest in obtaining large amounts of AfpB, so we assessed this possibility using our new TMV vector system. We designed two different constructs to target this protein to two different subcellular compartments, namely apoplast and endoplasmic reticulum (ER). Compartmentalizing antimicrobial peptides in plant cells is known to favor their accumulation by protecting them from host proteases and avoiding their toxic effects on the host cells (Bundó *et al*., 2014; Coca *et al*., 2006; Montesinos *et al*., 2016). To target AfpB to the apoplast or ER, we added the signal peptide of the tobacco AP24 protein (XP_009782398.1) to the amino terminus of the mature AfpB protein (Figure 2a). This signal peptide allows the protein to enter the secretory pathway toward the extracellular space. In the second construct, we added an additional KDEL sequence to the carboxyl terminus of the protein, facilitating retention inside the ER. We then expressed the corresponding recombinant viruses (TMVΔCP‐AfpB and TMVΔCP‐AfpBKDEL; Figure S4) in *N. benthamiana* leaves via infiltration with *A. tumefaciens* cultures. We observed that leaves agroinfiltrated with AfpB constructs were damaged, particularly those with the construct designed for AfpB accumulation in ER (Figure 2b). In contrast, leaves agroinfiltrated with a GFP control (TMVΔCP‐GFP) showed a healthy green appearance, similar to that of the mock‐agroinfiltrated leaves (Figure 2b). This suggests that the negative effects might be due to *afpB* expression rather than to TMVΔCP infection.

To confirm that AfpB was produced in *N. benthamiana* leaves, we extracted the apoplastic and total protein content, and analysed them by SDS‐PAGE. Coomassie blue staining showed the accumulation of a small protein with same apparent molecular weight to that of the *P. digitatum* AfpB (6.46 kDa) in the extracellular fluids (ECFs) of TMVΔCP‐AfpB agroinfiltrated leaves, but which was absent in leaves that had been agroinfiltrated with TMVΔCP‐GFP (Figure 2c). This protein was immunodetected with specific anti‐PAFB antibodies by Western blot analysis (Huber *et al*., 2018) (Figure 2d), thus demonstrating that AfpB was produced in *N. benthamiana* leaves when targeted to the apoplastic space.

We observed no differential bands in total acid protein extracts from leaves agroinfiltrated with TMVΔCP‐AfpBKDEL and mock inoculated leaves (Figure 2e). In contrast, GFP was observed clearly in the total protein extracts from TMVΔCP‐GFP leaves (Figure 2e). Moreover, Western blot analysis of total acid protein extracts did not detect AfpB in leaves inoculated with TMVΔCP‐AfpBKDEL, although the protein was detected in TMVΔCP‐AfpB leaves (Figure 2e). These observations indicate that AfpB did not accumulate in the ER vesicles of *N. benthamiana* leaf cells, probably due to toxic effects. By comparing with the known amounts of AfpB produced by *P. digitatum*, we estimated that the accumulation of AfpB in the ECFs was ~225 ± 37 μg per gram of *N. benthamiana* leaves. Thus, the recombinant protein accounts for more than 60% of the total ECF protein.

### Efficient production of proteins of fungal origin targeted to different subcellular compartments

**Figure 3:**
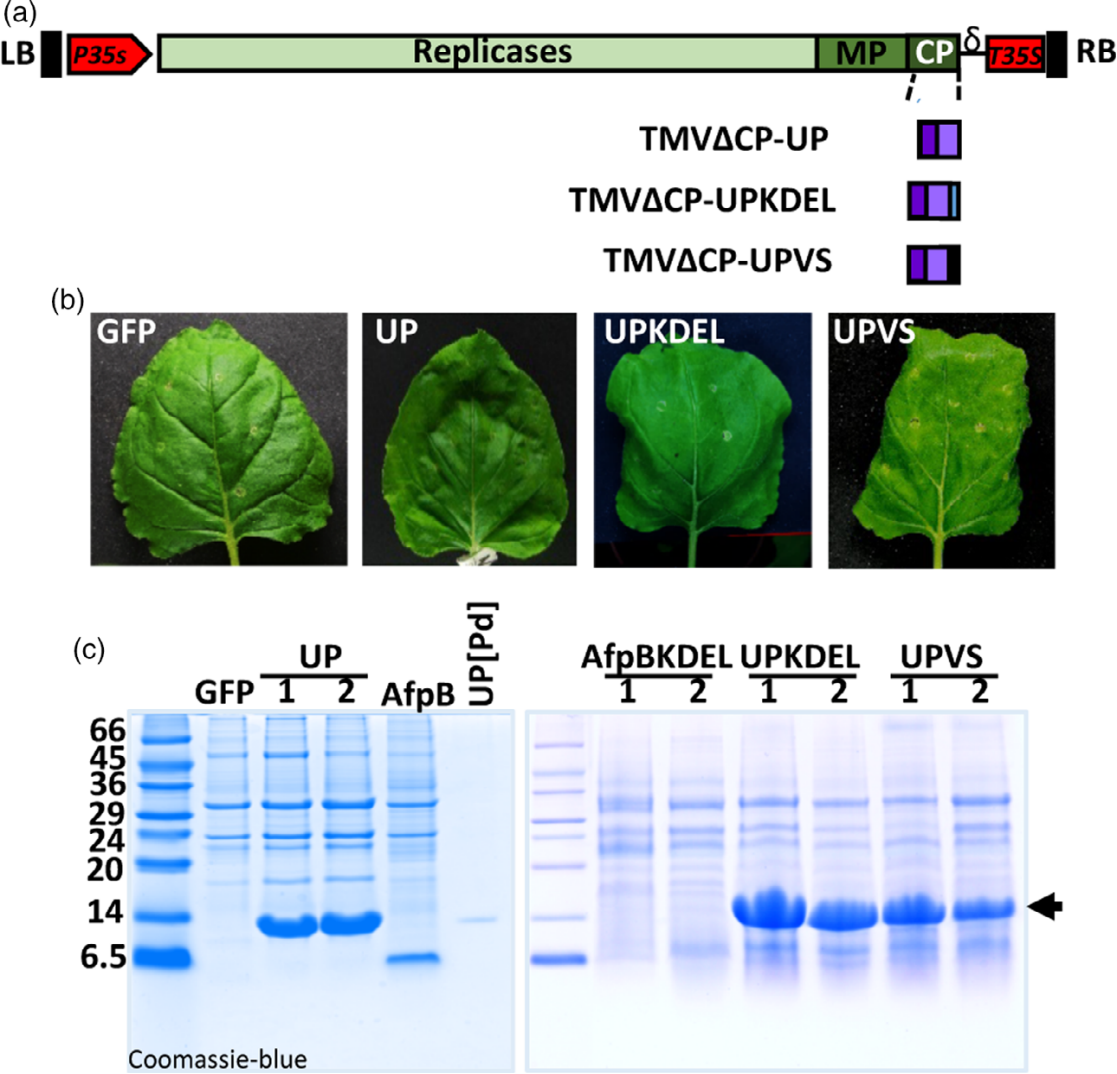
Production of large amounts of a *Penicillium digitatum* unknown protein in *Nicotiana benthamiana* leaves. (a) Schematic representation of the constructs generated to accumulate the protein of unknown function (UP, PDIG_23520) in the apoplast, ER, or vacuole of plant cells. The three constructs include the signal peptide of the tobacco AP24 protein at the N‐terminus. A KDEL signal was added at the C‐terminus in the construct designed for ER‐retention, and the vacuolar signal (VS) was fused at the C‐end for vacuolar accumulation. (b) Appearance of *N. benthamiana* leaves agroinfiltrated with the indicated constructs after 7 days. (c) Analysis of UP accumulation in agroinfiltrated leaves. Proteins from extracellular fluids (ECFs, left panel) or acid extraction (right panel) were separated by tricine‐SDS‐PAGE and Coomassie‐blue stained. Purified UP from *P. digitatum* cultures was run in parallel as a control. Molecular weight markers are shown in kDa on the left.

Since AfpB was not produced when targeted to the ER, we assessed the efficiency of the TMV‐based expression system using a different fungal protein and different subcellular localizations. We selected a protein of unknown function (UP, unknown protein) encoded by the genome of *P. digitatum* (PDIG_23520); UP is a small, cysteine‐rich secreted protein that has no predicted antifungal activity (Garrigues and Marcos, unpublished observations). Preliminary experiments showed that this protein was readily produced when secreted to the apoplast space (Figure 2c). We prepared three different constructs to target UP to the apoplast, the ER, or the vacuole (recombinant viruses TMVΔCP‐UP, TMVΔCP‐UPKDEL and TMVΔCP‐UPVS, respectively; Figures 3a and S4). The constructs to target UP to the apoplast and ER were prepared as described for AfpB. The vacuolar construct carries the same N‐terminal signal peptide, which allows UP to enter the secretory pathway, and also a vacuolar signal peptide at the carboxyl terminus of the protein (Neuhaus *et al*., 1991). *Nicotiana benthamiana* leaves agroinfiltrated with these recombinant viruses appeared normal with respect to the TMVΔCP‐GFP control (Figure 3b). We observed a protein with the expected mobility of *P. digitatum* UP in the ECFs obtained from TMVΔCP‐UP‐infiltrated leaves (Figure 3c, left panel). As judged from the intensity of bands stained with Coomassie blue, this protein achieved a higher level of accumulation than those reached by AfpB. Moreover, we obtained large amounts of this unknown protein when using TMVΔCP‐UPKDEL and TMVΔCP‐UPVS, the constructs designed for intracellular accumulation in ER vesicles or vacuoles (Figure 3c, right panel). These differences were striking, considering that AfpB was not detected when targeted to the ER. We estimated that the recombinant protein accumulated in the ECFs to a concentration of 4.3 ± 1.1 mg per gram of leaf fresh weight. These results demonstrate that the new TMV‐based protein production system is highly efficient, and allows one to easily target recombinant proteins to different subcellular compartments.

### The AfpB produced in plants is biologically active and indistinguishable from the produced by *P. digitatum*

**Figure 4:**
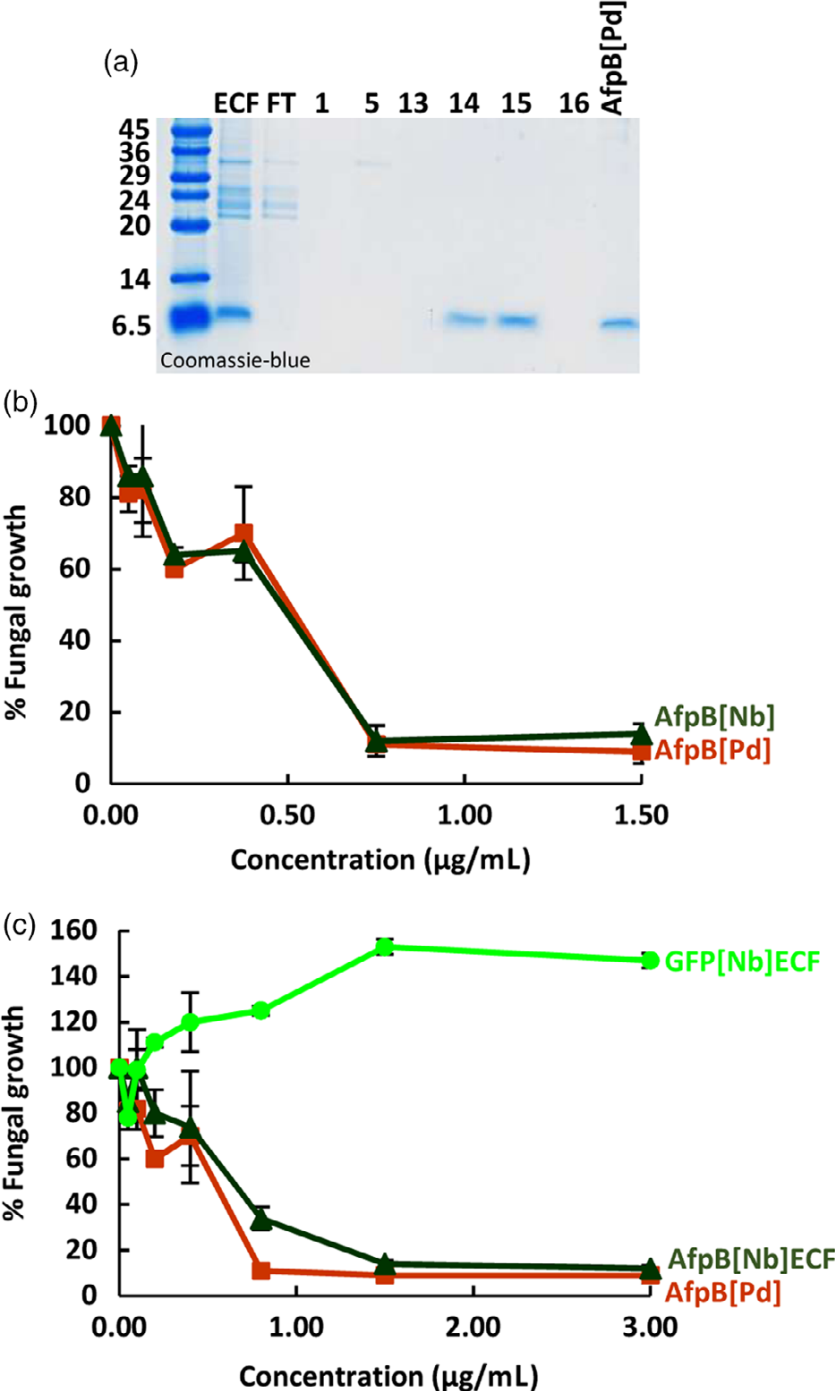
Equivalence of antifungal activity for the *in planta*‐and *in fungus*‐produced AfpB. (a) Purification of AfpB from *Nicotiana benthamiana* leaves by cationic exchange chromatography. Coomassie‐blue stained tricine‐SDS‐PAGE gel loaded with equivalent volumes of extracelular fluid (ECF), flow‐through (FT) and different eluted chromatographic fractions (1, 5, 13‐16), and 3.5 μg of *Penicillium digitatum* purified protein (AfpB[Pd]). (b‐c) *In vitro* inhibitory activity of AfpB against *P. digitatum* fungus. Dose–response curves comparing the fungal growth inhibitory activity of the purified AfpB[Pd] and *N. benthamiana* AfpB (AfpB[Nb]) (b), or with ECFs from leaves of *N. benthamiana* producing AfpB (AfpB[Nb] ECF) or GFP (GFP[Nb] ECF) (c). Data shown represent the mean ± SD of three biological replicates after 72 h of incubation with the indicated amounts of AfpB.

Having established that AfpB can be produced in plants when targeted to the extracellular space, we then proceeded to its purification from plant ECFs and to determine its biological activity. For purification, we subjected ECFs from agroinfiltrated *N. benthamiana* leaves to one‐step cation‐exchange chromatography. We found that most proteins in the extract, except for AfpB, were not retained by the cationic column, and were found in the flow‐through (Figure 4a). We then eluted the retained AfpB from the column as a single peak in fractions 14 and 15 at 0.25 M NaCl concentration, and detected a single protein band by SDS‐PAGE analysis. Next we compared the antifungal activity of the purified recombinant AfpB (AfpB[Nb]) to that of the AfpB produced by *P. digitatum* (AfpB[Pd]). The activity was assayed against the producer fungus *P. digitatum*, an economically relevant pathogen of citrus fruits against which the fungal protein showed potent activity (Garrigues *et al*., 2017). Growth inhibitory assays at different protein concentrations showed that the recombinant protein produced in biofactory plants had equivalent activity to the fungal version (Figure 4b). Importantly, the crude ECFs obtained from infiltrated plant tissues showed antifungal activity as result of AfpB production, and this activity was similar to that of the purified AfpB from fungal origin (Figure 4c). These results indicate that purification to homogeneity is not required in order to obtain a good AfpB activity.

### TMV‐based production of alternative antifungal proteins in biofactory plants

**Figure 5:**
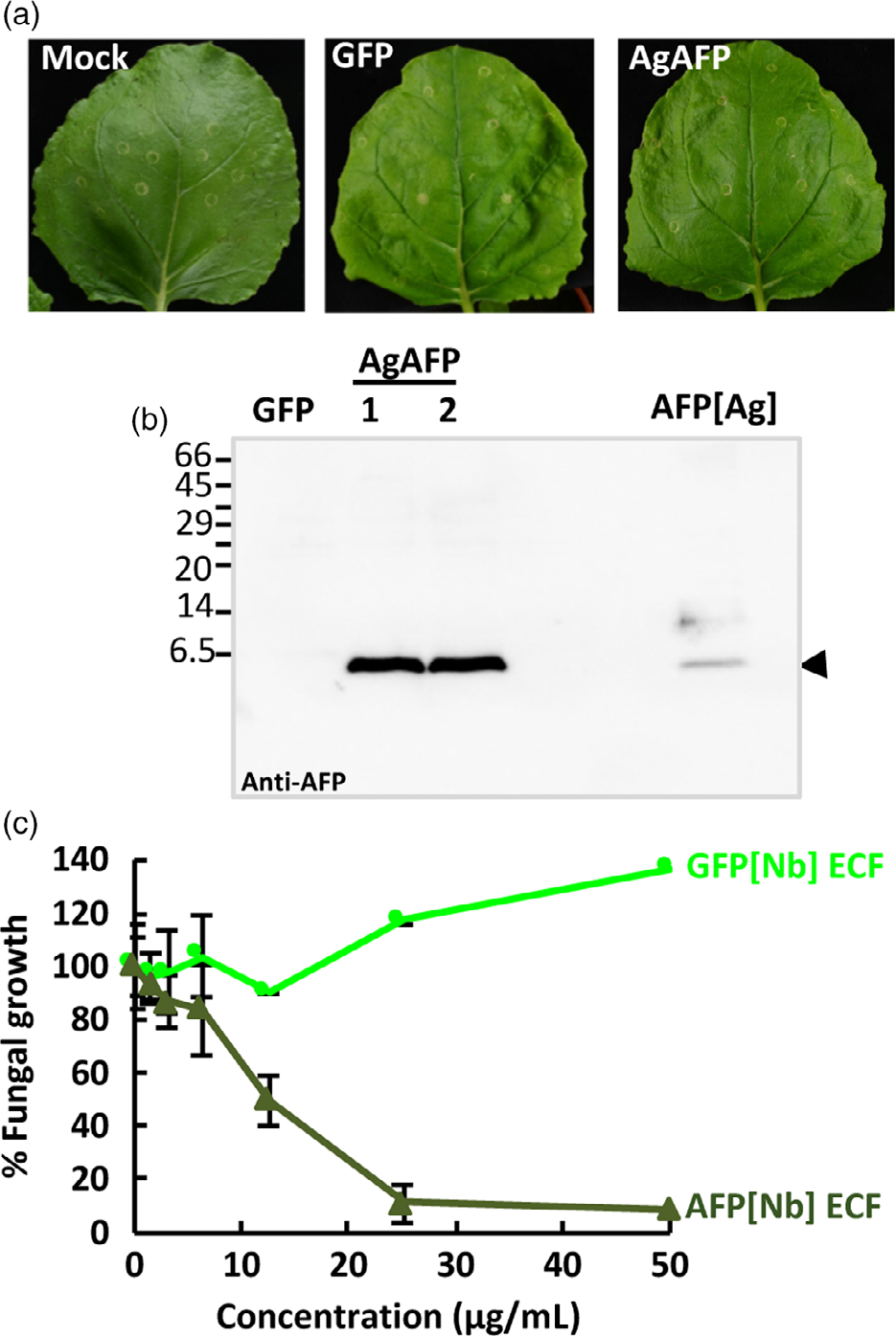
Production of *Aspergillus giganteus* AFP (AgAFP) in the apoplast of *Nicotiana benthamiana* leaves. (a) Appearance of *N. benthamiana* leaves 7 days after agroinoculation with the indicated constructs. (b) Analysis of AgAFP accumulation in the extracellular fluid (ECF) from two independent plants (1 and 2) by Western blot immunodetection using anti‐AFP‐specific antibodies. Purified AgAFP (250 ng) from *A. giganteus* cultures was run in parallel as a control (AFP[Ag]). Molecular weight markers are shown in kDa on the left. (c) Antifungal activity of ECFs containing AFP[Nb] against *Penicillium digitatum*. Dose–response curves of the fungal growth inhibitory activity of AFP[Nb] ECF in comparison to GFP[Nb] ECF. Data shown represent the mean ± SD of three biological replicates after 72 h of incubation with the indicated amounts of AFP.

Finally, we wanted to evaluate the new TMV‐based system with other antifungal proteins. For this, we chose the well‐characterized AFP from *Aspergillus giganteus* (Campos‐Olivas *et al*., 1995; Meyer, 2008; Vila *et al*., 2001). Based on the results obtained with the AfpB, we decided to directly target AgAFP accumulation to the apoplastic space. We generated the TMVΔCP‐AgAFP construct containing the sequence encoding the AP24 secretion signal peptide and the mature AgAFP (Figure S4). *Nicotiana benthamiana* leaves infiltrated with this construct had a similar appearance to the mock infiltrated leaves and leaves inoculated with the TMVΔCP‐GFP control (Figure 5a). We analysed ECFs from *N. benthamiana* leaves by Western blot using the specific antibodies against AgAFP, previously described (Coca *et al*., 2004), and found that AgAFP accumulated in the apoplasts of infiltrated leaves (Figure 5b). By comparison to known amounts of the fungal protein, we estimated the amount of protein produced to be around 196 ± 72 μg per gram of leaf fresh weight. Moreover, ECFs enriched in AgAFP showed antifungal activity against *P. digitatum* (Figure 5c). This activity seems to be at least one order of magnitude lower than that of the ECFs containing AfpB (Figure 4c), in agreement with the high activity reported for AfpB (Garrigues *et al*., 2017). These results, obtained with a different AFP, indicate that our TMV‐based system can be considered a general platform for producing antifungal proteins in biofactory plants.

### Optimization of the AfpB production method for scaling the process

**Figure 6:**
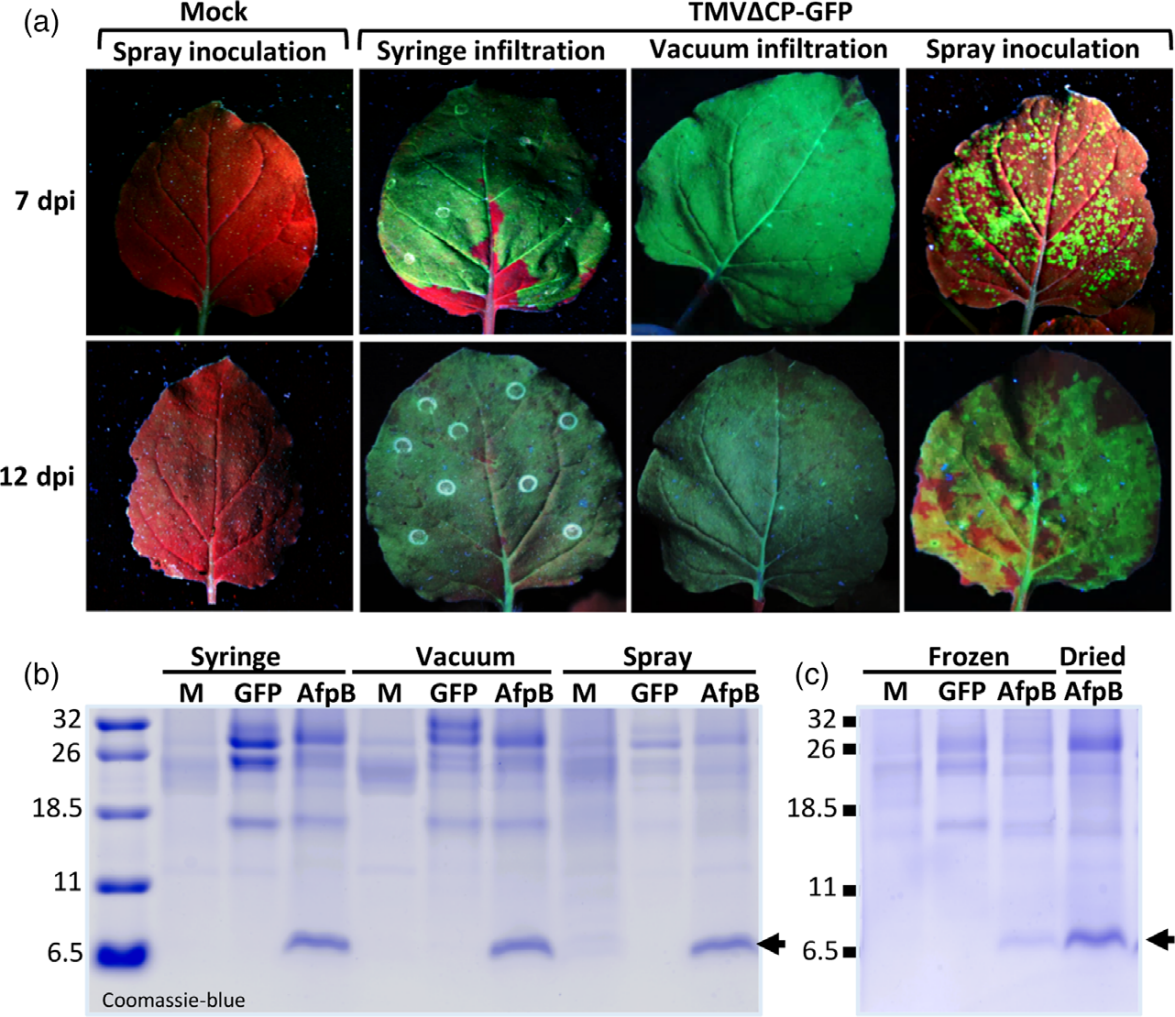
Optimization of AfpB production method for scaling the process. (a) Efficacy of different agroinoculation methods using TMVΔCP‐GFP. Representative *Nicotiana benthamiana* leaves visualized under UV light at 7 or 12 days postinoculation (dpi), as indicated. (b) Comparative production of AfpB by agroinoculation with TMVΔCP‐AfpB using the indicated methods. Coomassie‐blue staining of ECF proteins from leaves at 12 dpi (c) Stability of AfpB upon dried storage of agroinoculated leaves. Coomassie‐blue staining of total protein extracts obtained from leaves that were immediately frozen or dry‐stored for 2 months.

Syringe inoculation of plants is a time‐consuming and labor intense process, and thus not suitable for large‐scale production of AFPs. We evaluated simple methods for agroinoculation that could facilitate and speed up the AFP production process. These methods included vacuum infiltration, which can be done on a large scale with robotics, and simpler spray applications, as reported previously (Hahn *et al*., 2015). To monitor transfection efficiency easily, we used the construct TMVΔCP‐GFP to produce GFP that can be visualized under UV light. In syringe‐ or vacuum‐agroinfiltrated leaves, we observed a strong and homogeneous distribution of GFP fluorescence in the leaves at 7 days, while in sprayed leaves we could distinguish separate GFP foci (Figure 6a, upper panels). However, these GFP foci enlarged over time and merged at 12 days (Figure 6a, lower panels). These observations indicate that all three methods can efficiently produce GFP, although the spray application method requires a longer timeframe. We also tested these methods for AfpB production, and found that ECFs from agroinoculated leaves showed similar AfpB accumulation at 12 days for all three methods (Figure 6b). These results indicate that the much simpler spray application can be implemented for easy and inexpensive large‐scale AfpB production.

Finally, we evaluated the stability of the *in planta* accumulated AfpB during storage as dried material, which could allow producers to uncouple biomass production from processing. Comparing AfpB accumulation in dried leaves with that in immediately frozen leaves, we observed that AfpB remains stable at least for 2 months when stored in dried leaves (Figure 6c). Therefore, AfpB can be manufactured in *N. benthamiana* without the need for immediate processing, and can be stored in dried leaves.

### Protection of tomato plants against Botrytis grey mold by *in planta*‐produced AfpB

**Figure 7:**
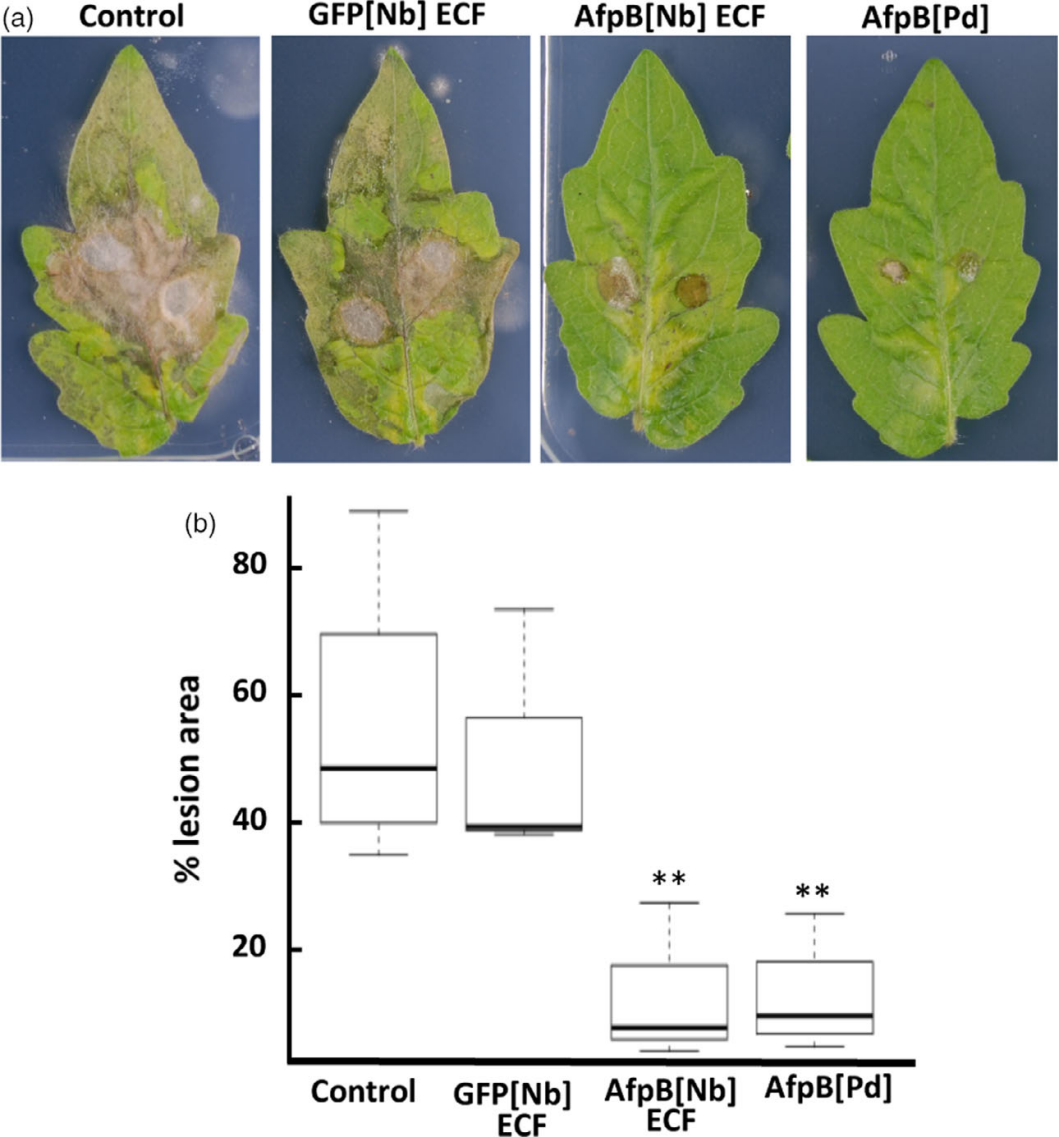
Protection against *Botrytis cinerea* infection on tomato leaves by *in planta‐*produced AfpB produced in *Nicotiana benthamiana*. Representative leaves at 4 days after drop‐inoculation with *B*. conidia suspensions (0.5 × 10^6^ conidia/mL) along with *N. benthamiana* ECFs containing AfpB (10 μm AfpB[Nb]), GFP ECFs (GFP[Nb]) or fungal protein (10 μm AfpB[Pd]). (b) Box plot of the percentage of leaf damage area from the indicated treatments. Data represent three independent experiments. Asterisks denote statistical significance (Tukey test, **P < 0.05).

Finally, we assessed the effectiveness of *in planta*‐produced AfpB in plant protection assays. For that, plant protein extracts containing AfpB were evaluated against the broad‐spectrum fungal pathogen *B. cinerea*. This fungus causes gray mold in plants and fresh fruits and vegetables, and is responsible for important economic losses (Dean *et al*., 2012). AfpB produced by *P. digitatum* has *in vitro* inhibitory activity against *B. cinerea* with a MIC (minimal inhibitory concentration) value of 12.5 μm (Garrigues *et al*., 2017). We deposited drops containing fungal conidia on tomato leaves along with *N. benthamiana* ECF containing 10 μm AfpB, as well as, drops with conidia along with the same concentration of pure AfpB produced by *P. digitatum,* and control drops with conidia along with water or ECF from GFP producing plants. Where control drops were deposited, infection symptoms were clearly visible at 4 days postinoculation (dpi); whereas where drops containing AfpB were deposited lesions were clearly smaller (Figure 7a). By quantifying lesion size using image analysis, we observed a statistically significant decrease in the damaged area in the presence of AfpB (Figure 7b). These results demonstrate that AfpB protects against *B. cinerea* infection, both for crude ECFs containing AfpB (AfpB[Nb]ECF), and for AfpB purified from fungal cultures (AfpB[Pd]). Even more interesting, the ECF extracts containing AfpB showed the same protective efficacy. Therefore, depending on the intended use for the AfpB protein, it may not be necessary to include a downstream purification process. This would reduce considerably the costs of production and biotechnological application of this antifungal biomolecule.

## Discussion

In this study, we show that *N. benthamiana* plants are an excellent biofactory for producing antifungal proteins of fungal origin when transiently expressed using a TMV‐derived vector. The process we have developed is fast and produces high yields of antifungal proteins, which, in addition, are bioactive in the crude ECF, or can be easily purified from crude extracts. Importantly, the recombinant proteins produced in plants have exactly the same antifungal activity as their native counterparts purified from fungi. This is a remarkable result because these small cysteine‐rich proteins have been shown to be difficult to produce in a soluble, correctly folded conformation in bacterial systems (Kiedzierska *et al*., 2008; Rosano and Ceccarelli, 2014). They only had been successfully produced as active proteins in *Pichia pastori* at relatively low yields (Garrigues *et al*., 2017; López‐García *et al*., 2010; Virágh *et al*., 2014), and at better yields in filamentous fungi (Garrigues *et al*., 2017; Huber *et al*., 2018; Sonderegger *et al*., 2016; Tóth *et al*., 2018). Although plant and fungal systems are similar in terms of yield and activity, the transient production of AFPs in plants represents an attractive alternative as sustainable bioproduction factories fueled by sunlight and easily scalable. The developments reported here expand the available alternatives for the exploitation of antifungal proteins and other cysteine‐rich proteins such as UP, which was produced to extremely high yields.

To produce these antifungal proteins in plants, we first developed a new TMV‐based vector system. The main reason for developing another TMV vector was to exploit the Gibson assembly reaction to insert the ORFs of interest into the viral genome. The Gibson assembly reaction, which is extremely efficient for inserting DNA fragments into plasmids, provides unprecedented yields when assembling several DNA fragments into a plasmid in a single step, provided these fragments contain the appropriate overlapping ends (Gibson *et al*., 2009). This reaction facilitates the insertion of tags and the fusion of protein moieties. Moreover, we feel that modular TMV‐derived systems that re‐assemble *in vivo* (Marillonnet *et al*., 2004, 2005) are unnecessarily complex, and moreover they are not freely available to the scientific community. Here, starting with an infectious clone of a TMV vulgare strain (GenBank accession number MK087763) and a small, optimized binary vector (Thole *et al*., 2007), we constructed the plasmid pGTMV, which mediates the fast and efficient infection of *N. benthamiana* plants after infiltration with transformed *A. tumefaciens*. Marillonnet *et al*. (2005) suggested that plant agroinoculation with TMV is an inefficient process, attributed to abnormal RNA processing of the viral genome after transcription in the cellular nucleus, a step that is certainly alien to the natural TMV replication cycle. However, our experimental results demonstrate high infectivity, even at low densities of *A. tumefaciens* (Figure S5). The low infectivity previously reported in agroinoculation experiments may have been due to the use of inefficient genetic elements such as promoters and ribozymes, or the use of large, unstable binary plasmids.

Several plant‐based systems have been used to produce small proteins and peptides with antimicrobial activity. While challenging due to the physicochemical properties and toxicity of many of these peptides, some of these platforms successfully produce AMPs. These platforms include leaf chloroplasts (Lee *et al*., 2011), rice seeds (Bundó *et al*., 2014; Montesinos *et al*., 2016, 2017), barley seeds (Holásková *et al*., 2018) and hairy roots (Chahardoli *et al*., 2018). However, the yields reported when using these systems were much lower than the AFP yield achieved in *N. benthamiana* using the TMV‐derived system reported here. While our estimated yield of AfpB was approximately 250 μg per gram of fresh *N. benthamiana* leaf tissue, the reported yields for Retrocyclin‐101 and Protegrin‐1 were ~5 and 8 μg/g of tobacco respectively (Lee *et al*., 2011), those for Cecropin A were ~40 μg/g of rice seeds (Montesinos *et al*., 2016), those for LL37 were ~0.55 μg/g of barley seeds (Holásková *et al*., 2018), and those for Lactoferrin chimera were ~4.8 μg/g of tobacco hairy roots (Chahardoli *et al*., 2018). In addition, all of these plant‐based production systems are based on stable transformation, which makes the process more complex and time consuming. Other transient expression systems have also been used for AMP production, although with lower yields than those we achieved for AFPs (Patiño‐Rodríguez *et al*., 2013; Zeitler *et al*., 2013). Zeitler *et al*. (2013) used a TMV full‐length virus strategy, to produce recombinant linear AMP SP1‐1 at a yield of ~25 μg/g of *N. benthamiana* leaf tissue, while Patiño‐Rodríguez *et al*. (2013) used the MagnICON system to produce Protegrin‐1 at lower amounts than for the AFPs, and without providing quantitative data. In any case, even our yield of 250 μg/g for AfpB is far from the 4 mg/g achieved for other proteins, such as GFP or the *P*. *digitatum* unknown protein UP, which has no predicted antimicrobial activity. This highlights the difficulties associated with producing bioactive peptides in biofactory plants.

Importantly, our results with AfpB show that the subcellular localization is relevant to achieve high accumulations of bioactive peptides in plant tissues. AfpB accumulates at high levels when targeted to the extracellular space, whereas intracellular accumulation is hindered, probably by toxic effects. This observation was somehow unexpected because AFP has not previously been found to be toxic in plant cells (Coca *et al*., 2004; Girgi *et al*., 2006; Moreno *et al*., 2006, 2005; Oldach *et al*., 2001; Vila *et al*., 2001). However, these previous studies used external application or extracellular localization of AFPs in plant tissues, so it is possible that AFPs interfere with plant cell functions once inside the cells. In contrast, the other fungal protein produced in our experiments, UP, which has no predicted antifungal activity, accumulated to similarly high intracellular and extracellular levels. These observations reveal the importance of targeting protein accumulation depending on the characteristics and activity of the recombinant protein.

Additionally, apoplastic targeting of AFPs in plant tissues greatly facilitates their purification. As observed in our work, an extremely simple purification scheme is required to purify AFPs from *N. benthamiana* tissue. ECFs can be obtained easily by vacuum infiltration of harvested leaves and sieving, yielding highly AFP‐rich solutions, in which more than half of the protein content is AFPs. Starting with these ECFs, a single chromatographic step is required to purify these small proteins to homogeneity. Even better, our results show that ECFs can be used directly as antifungals with no further purification. Thus, the downstream processing of recombinant AFPs can be greatly reduced and, consequently, the overall manufacturing costs.

In addition to leaf infiltration, we have also shown that the TMV‐derived system can be used to produce AFPs by spraying *N. benthamiana* tissues with *A. tumefaciens* suspensions. This simplification of the inoculation process was previously reported for the MagnICON system (Hahn *et al*., 2015), and we have successfully implemented it for our new TMV‐derived system. We demonstrate that the AFP yields achieved after 12 days by spraying leaves are similar to those achieved after 7 days by vacuum or syringe infiltration, although spraying is a much simpler inoculation strategy. Moreover, we observed that AFPs remain stable in stored *N. benthamiana* leaves for at least 2 months at room temperature. This observation suggests that the AFP production process can be decoupled from the purification process, and leaves can be conveniently stored before purification. There are clear advantages to decoupling these two processes, such as avoiding immediate processing after harvesting or otherwise freezing the plant material, with their associated production costs. These developments facilitate large‐scale production of these proteins in an easy, fast cost‐efficient and safe manner. In our TMV vector, the viral CP is replaced with the AFP genes, so no virus particles are created, and therefore there is no risk of viral dissemination in the environment. Also, the *afp* genes are not incorporated into the plant genome so they did not become heritable traits. Nonetheless, industrial production would require contained greenhouse conditions to avoid the release of the genetically modified *A. tumefaciens* strains and TMV clones. The green AFP manufacturing process developed here could contribute to bring these proteins into the market for practical use as antifungals. To support these applications, we also demonstrated the effectiveness of plant ECF containing AfpB in protecting tomato plants against Botrytis grey mold. *Botrytis cinerea* is one of the top ten fungal phytopathogens, causing important economic losses due to the broad range of hosts, and that damage that can occur during the production and postharvesting of vegetables, fruits and flowers (Dean *et al*., 2012). Fungicides are the commonly used method for controlling *B. cinerea*, and botryticides represent more than 10% of the world's fungicide market. This work supports the use of green AfpB as an environment friendly and sustainable alternative to chemical fungicides.

## Experimental procedures

### Plasmid construction

To build pGTMV, pMTMVi‐N and pMTMVi‐M (Addgene plasmids # 118755, 118756 and 118757, respectively), PCR amplification reactions were performed using the Phusion high‐fidelity DNA polymerase (Thermo Scientific) in HF buffer. The Gibson assembly reactions were performed using the NEBuilder HiFi DNA assembly master mix (New England Biolabs). The NEBuilder assembly tool (https://nebuilder.neb.com/; New England Biolabs) was used to design the primers. *Escherichia coli* DH5α were electroporated with the products of the Gibson assembly reaction. The full sequence of these plasmids, which was confirmed experimentally by nucleotide sequencing, is shown in Figure S2. To build plasmids expressing the various recombinant TMV clones used in this work, the intermediate plasmids pMTMVi‐N or pMTMVi‐M were digested with *Age*I and *Xho*I (Thermo Scientific), and the DNA fragments corresponding to the ORFs of interest were obtained by PCR and assembled using the Gibson reaction. Next, the manipulated fragment of the TMV cDNA was excised from the resulting plasmids by digestion with *Bsa*I (New England Biolabs), and assembled with the large fragment of pGTMV digested with *Nco*I‐*Pfl*23II (pMTMVi‐N derivatives) or *Nco*I‐*Cpo*I (pMTMVi‐M derivatives) (Thermo Scientific). The full sequence of all recombinant viruses is shown in Figure S4.

### Agroinoculation of *N. benthamiana* leaves

*Agrobacterium tumefaciens* GV3101 carrying the helper plasmid pSoup (Hellens *et al*., 2000) was transformed with plasmids to express the different TMV recombinant clones. For plant agroinoculation, overnight cultures of *A. tumefaciens* were diluted in induction medium (10 mm MES, 10 mm MgCl_2,_ 200 μm acetosyringone) at the appropriate optical density at 600 nm (OD_600_), incubated for 3 h at room temperature, and infiltrated using a needle‐less syringe (0.5 OD_600_) or sprayed on the underside of leaves using an aerograph at 2 atmospheres of pressure (1.0 OD_600_). Importantly, the surfactant Silwet L‐77 (0.1% v/v) needs to be added to the induction medium in the spray inoculation experiments for the effective agroinoculation. *N. benthamiana* plants were grown in the greenhouse at 24 °C with a 14 h light‐10 h dark photoperiod. For the inoculation experiments, we used plants at the 4‐ to 5‐leaf stage, without visible flower buds. Unless indicated in the results, leaves were harvested at 7 dpi or 12 dpi and examined for protein production. In storage experiments, leaves were dried in an incubator at 37 °C for 2 months. Plants accumulating the GFP were photographed at different time points with a Nikon D7000 digital camera under illumination with a hand‐held UV lamp.

### Protein analysis and purification

Protein samples were prepared by freezing agroinfiltrated leaves in liquid nitrogen and then grinding to a fine power with a mortar and pestle. Total soluble proteins were obtained in two volumes of 50 mm sodium phosphate buffer pH 7.2, 10 mm EDTA, 10 mm DTT, 0.1% (w/v) SDS and 0.1% (v/v) Triton X‐100. Extracts were clarified by centrifugation at 16000 g for 15 min at 4 °C. Acidic extraction was used with AFP samples, as these proteins are pH‐stable, and can be selectively extracted at low pH. The acidic buffer (pH 2.8) contained 84 mm citric acid, 30 mm Na_2_HPO_4_, 6 mm ascorbic acid, 0.1% (v/v) 2‐mercaptoethanol. For dried leaves, total proteins were extracted in 50 mm sodium acetate (pH 5.5), 100 mm NaCl, and 10% (v/v) glycerol. Apoplastic fluids were extracted from fresh leaves by vacuum infiltration using phosphate buffered saline (PBS) buffer supplemented with 0.02% (v/v) Silwet L‐77. Protein concentrations were determined using the Bio‐Rad protein assay and bovine serum albumin (BSA) as standard.

Protein preparations were separated in tricine‐SDS‐PAGE (16.5%). Gels were stained with Coomassie blue to detect proteins, or transferred to nitrocellulose membranes (Protran 0.2 μm) to immunodetect proteins, as described previously (Coca *et al*., 2004; Garrigues *et al*., 2017). To detect AfpB, we used antiserum against *P. chrysogenum* PAFB, which was kindly provided by Dr. Florentine Marx (Medical University of Innsbruck, Austria) (Huber *et al*., 2018). AFPs were purified to homogeneity by cationic chromatography using an AKTA Purifier system equipped with a 5 mL HiTrap SP HP column (GE Healthcare), as described previously (Garrigues *et al*., 2017). AfpB from *P. digitatum* (AfpB[Pd]) was obtained from the genetically modified strain PDSG3543 and purified from culture supernatants using the same cationic chromatography (Garrigues *et al*., 2017). Protein concentrations were determined spectrophotometrically at 280 nm in purified fractions, or by comparing the band intensities to known amounts of purified proteins in complex protein extracts, such as extracellular fluids. Signal intensities were quantified using the Quantity Tools Image Lab™ Software (Version 5.2.1) included in the ChemiDoc™ Touch Imaging System (Bio‐Rad). The percentage of the recombinant protein on the total ECF protein concentration was then calculated. GFP fluorescence in protein extracts was determined as described previously (Lindbo, 2007).

### Antifungal assays

Growth inhibition assays of the *P. digitatum* CECT20796 (PHI26) strain were performed in 96‐well microtiter plates, as described previously (Garrigues *et al*., 2017). Purified proteins and apoplastic fluids were dialysed against water and used at the concentrations indicated in antifungal assays.

### Plant protection assays

*Botrytis cinerea* was kindly provided by Prof. A. Molina (CBGP collection, Madrid). Tomato plants (*Solanum lycopersicum* cultivar Marmande, known as the Mediterranean tomato) were cultivated in growth chambers at 22 °C with a 16 h light‐8 h dark photoperiod. AfpB protection assays against *Botrytis cinerea* infection were performed on detached tomato leaves of 3‐week‐old plants on 1% (w/v) agar in water containing 2 μg/mL kinetin. Leaves were locally infected at two points with conidial suspensions (10^6^ conidia/mL) by applying 20 μL drops containing 10 μm AfpB of purified protein or extracellular fluids. The progression of symptoms was followed visually. Lesion area was measured by image analysis using the Fiji ImageJ2 package. We analysed two infection points on three leaves from three independent plants in three independent experiments.

## Conflict of interest

The authors declare no conflict of interest.

## Supporting information


**Figure S1** TMV infectious clone.
**Figure S2** Full sequences of pGTMV, pMTMVi‐N and pMTMVi‐M plasmids.
**Figure S3** Analysis of GFP produced in *Nicotiana benthamiana* plants using the TMV‐derived system.
**Figure S4** Sequence of TMV‐derived recombinant viruses TMVΔCP‐GFP, TMV(ToCP)‐fGFP, TMVΔCP‐AfpB, TMVΔCP‐AfpBKDEL, TMVΔCP‐UP, TMVΔCP‐UPKDEL, TMVΔCP‐UPVS, and TMVΔCP‐AgAFP.
**Figure S5** Dilution analysis of TMV∆CP‐GFP infectivity in *Nicotiana benthamiana*.
**Table S1** Primers used in this work.
