# Supplementary material for: Efficient production of antifungal proteins in plants using a new transient expression vector derived from tobacco mosaic virus
Source: Plant Biotechnol J. 2018 Dec 6;17(6):1069–80. doi: 10.1111/pbi.13038 (PMC6523586; doi:10.1111/pbi.13038)
Supplement: Supplementary file 1 — Figure S1 TMV infectious clone. Figure S2 Full sequences of pGTMV, pMTMVi‐N and pMTMVi‐M plasmids. Figure S3 Analysis of GFP produced in Nicotiana benthamiana plants using the TMV‐derived system. Figure S4 Sequence of TMV‐derived recombinant viruses TMVΔCP‐GFP, TMV(ToCP)‐fGFP, TMVΔCP‐AfpB, TMVΔCP‐AfpBKDEL, TMVΔCP‐UP, TMVΔCP‐UPKDEL, TMVΔCP‐UPVS, and TMVΔCP‐AgAFP. Figure S5 Dilution analysis of TMV∆CP‐GFP infectivity in Nicotiana benthamiana. Table S1 Primers used in this work. [file PBI-17-1069-s001.pdf]

SUPPORTING INFORMATION

Efficient production of antifungal proteins in plants using a new transient expression vector derived from tobacco mosaic virus

Xiaoqing Shi[1§], Teresa Cordero[2§], Sandra Garrigues[3], Jose F. Marcos[3], José-Antonio Daròs[2*], and María Coca[1*]

[1]*Centre for Research in Agricultural Genomics (CRAG, CSIC-IRTA-UAB-UB), 08193 Cerdanyola del Vallès, Spain*

[2]*Instituto de Biología Molecular y Celular de Plantas (IBMCP, CSIC-Universitat Politècnica de València), 46022 Valencia, Spain*

[3]*Instituto de Agroquímica y Tecnología de Alimentos (IATA, CSIC), 46980 Paterna, Spain*

[§]X.S. and T.C. contributed equally to this work.

*Correspondence (email: jadaros@ibmcp.upv.es, maria.coca@cragenomica.es)

Running title: A new viral system for efficient AFP production

Keywords: antifungal proteins, viral vector, tobacco mosaic virus, plant biofactory, *Nicotiana benthamiana,* Gibson assembly.

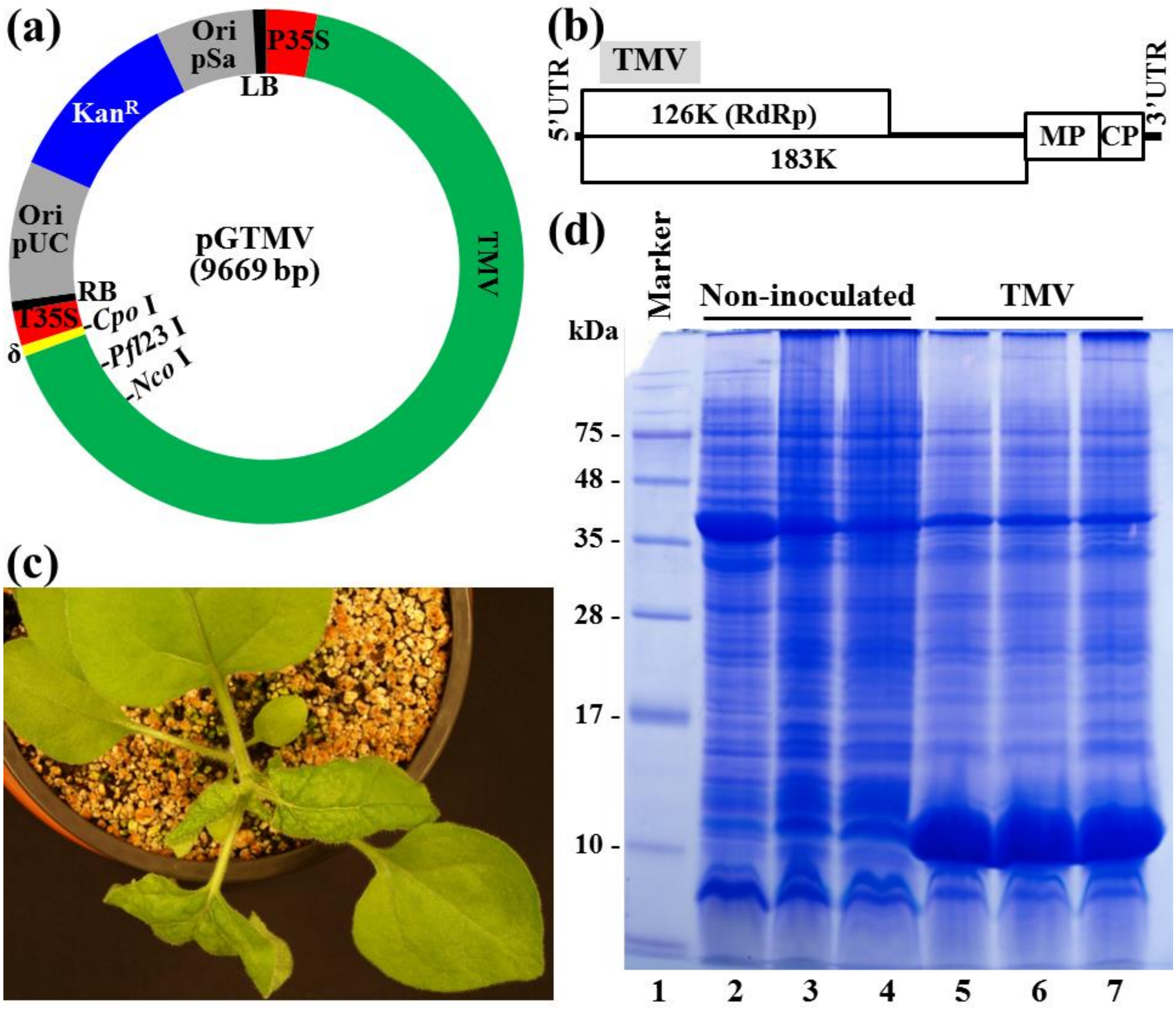

Figure S1. *Tobacco mosaic virus* (TMV) infectious clone. (a) Schematic representation of plasmid pGTMV that contains the whole TMV cDNA. See more details in the legend to Figure 1. (b) Schematic representation of wild-type TMV genome. Boxes represent the 126K and 183K replication proteins, movement protein (MP) and coat protein (CP). Lines represent the 5' and 3' untranslated regions (UTRs). (c) *Nicotiana benthamiana* plant infected with wild-type TMV. Picture was taken 5 days post-inoculation (dpi). (d) Coomassie blue-stained polyacrylamide gel showing the protein content of *N. benthamiana* leaves from plants mock-inoculated (lanes 2 to 4) or agroinoculated with TMV (lanes 5 to 7). Lane 1, marker proteins with sizes in kDa indicated on the left.

Figure S2. Full sequences of plasmid pGTMV, which contains a full-length TMV infectious clone (GenBank accession number MK087763), and intermediate plasmids pMTMVi-N and pMTMVi-M, which can be used to construct recombinant TMV clones unable and able to move systemically throughout the plant, respectively.

>pGTMV (9669 bp)
GCGGCCGC**GATTCCATTGCCCAGCTATCTGTCACTTTATTGTGAAGATAGTGGAAAAGGAAGGTGGCTCCTACAA**
ATGCCATCATTGCGATAAAGGAAAGGCCATCGTTGAAGATGCCTCTGCCGACAGTGGTCCCAAAGATGGACCCCC
ACCCACGAGGAGCATCGTGGAAAAAGAAGACGTTCCAACCACGTCTTCAAAGCAAGTGGATTGATGTGATATCTC
CACTGACGTAAGGGATGACGCACAATCCCACTATCCTTCGCAAGACCCTTCCTCTATATAAGGAAGTTCATTTCA
TTTGGAGAGGTATTTTTACAACAATTACCAACAACAACAAACAACAAACAACATTACAATTACTATTTACAATTA
CAATGGCATACACACAGACAGCTACCACATCAGCTTTGCTGGACACTGTCCGAGGAAACAACTCCTTGGTCAATG
ATCTAGCAAAGCGTCGTCTTTACGACACAGCGGTTGAAGAGTTTAACGCTCGTGACCGCAGGCCCAAGGTGAACT
TTTCAAAAGTAATAAGCGAGGAGCAGACGCTTATTGCTACCCGGGCGTATCCAGAATTCCAAATTACATTTTATA
ACACGCAAAATGCCGTGCATTCGCTTGCAGGTGGATTGCGATCTTTAGAACTGGAATATCTGATGATGCAAATTC
CCTACGGATCATTGACTTATGACATAGGCGGGAATTTTGCATCGCATCTGTTCAAGGGACGAGCATATGTACACT
GCTGCATGCCCAACCTGGACGTTCGAGACATCATGCGGCACGAAGGCCAGAAAGACAGTATTGAACTATACCTTT
CTAGGCTAGAGAGAGGGGGGAAAACAGTCCCCAACTTCCAAAAGGAAGCATTTGACAGATACGCAGAAATTCCTG
AAGACGCTGTCTGTCACAATACTTTCCAGACATGCGAACATCAGCCGATGCAGCAATCAGGCAGAGTGTATGCCA
TTGCGCTACACAGCATATATGACATACCAGCCGATGAGTTCGGGGCGGCACTCTTGAGGAAAAATGTCCATACGT
GCTATGCCGCTTTCCACTTCTCCGAGAACCTGCTTCTTGAAGATTCATGCGTCAATTTGGACGAAATCAACGCGT
GTTTTTCGCGCGATGGAGACAAGTTGACCTTTTCTTTTGCATCAGAGAGTACTCTTAATTACTGTCATAGTTATT
CTAATATTCTTAAGTATGTGTGCAAAACTTACTTCCCGGCCTCTAATAGAGAGGTTTACATGAAGGAGTTTTTAG
TCACCAGAGTTAATACCTGGTTTTGTAAGTTTTCTAGAATAGATACTTTTCTTTTGTACAAAGGTGTGGCCCATA
AAAGTGTAGATAGTGAGCAGTTTTATACTGCAATGGAAGACGCATGGCATTACAAAAAGACTCTTGCAATGTGCA
ACAGCGAGAGAATCCTCCTTGAGGATTCATCATCAGTCAATTACTGGTTTCCCAAAATGAGGGATATGGTCATCG
TACCATTATTCGACATTTCTTTGGAGACTAGTAAGAGGACGCGCAAGGAAGTCTTAGTGTCCAAGGATTTCGTGT
TTACAGTGCTTAACCACATTCGAACATACCAGGCGAAAGCTCTTACATACGCAAATGTTTTGTCCTTCGTCGAAT
CGATTCGATCGAGGGTAATCATTAACGGTGTGACAGCGAGGTCCGAATGGGATGTGGACAAATCTTTGTTACAAT
CCTTGTCCATGACGTTTTACCTGCATACTAAGCTTGCCGTTCTAAAGGATGACTTACTGATTAGCAAGTTTAGTC
TCGGTTCGAAAACGGTGTGCCAGCATGTGTGGGATGAGATTTCGCTGGCGTTTGGGAACGCATTTCCCTCCGTGA
AAGAGAGGCTCTTGAACAGGAAACTTATCAGAGTGGCAGGCGACGCATTAGAGATCAGGGTGCCTGATCTATATG
TGACCTTCCACGACAGATTAGTGACTGAGTACAAGGCCTCTGTGGACATGCCTGCGCTTGACATTAGGAAGAAGA
TGGAAGAAACGGAAGTGATGTACAATGCACTTTCAGAATTATCGGTGTTAAGGGAGTCTGACAAATTCGATGTTG
ATGTTTTTTCCCAGATGTGCCAATCTTTGGAAGTTGACCCAATGACGGCAGCGAAGGTTATAGTCGCGGTCATGA
GCAATGAGAGCGGTCTGACTCTCACATTTGAACGACCTACTGAGGCGAATGTTGCGCTAGCTTTACAGGATCAAG
AGAAGGCTTCAGAAGGTGCATTGGTAGTTACCTCAAGAGAAGTTGAAGAACCGTCCATGAAGGGTTCGATGGCCA
GAGGAGAGTTACAATTAGCTGGTCTTGCTGGAGATCATCCGGAGTCGTCCTATTCTAAGAACGAGGAGATAGAGT
CTTTAGAGCAGTTTCATATGGCGACGGCAGATTCGTTAATTCGTAAGCAGATGAGCTCGATTGTGTACACGGGTC
CGATTAAAGTTCAGCAAATGAAAAACTTTATCGATAGCCTGGTAGCATCACTATCTGCTGCGGTGTCGAATCTCG
TCAAGATCCTCAAAGATACAGCTGCTATTGACCTTGAAACCCGTCAAAAGTTTGGAGTCTTGGATGTTGCATCTA
GGAAGTGGTTAATCAAACCAACGGCCAAGAGTCATGCATGGGGTGTTGTTGAAACCCACGCGAGGAAGTATCATG
TGGCGCTTTTGGAATATGATGAGCAGGGTGTGGTGACATGCGATGATTGGAGAAGAGTAGCTGTTAGCTCTGAGT
CTGTTGTTTATTCCGACATGGCGAAACTCAGAACTCTGCGCAGACTGCTTCGAAACGGAGAACCGCATGTCAGTA
GCGCAAAGGTTGTTCTTGTGGACGGAGTTCCGGGCTGTGGAAAAACCAAAGAAATTCTTTCCAGGGTTAATTTTG
ATGAAGATCTAATTTTAGTACCTGGGAAGCAAGCCGCGGAAATGATCAGAAGACGTGCGAATTCCTCAGGGATTA
TTGTGGCCACGAAGGACAACGTTAAAACCGTTGATTCTTTCATGATGAATTTTGGGAAAAGCACACGCTGTCAGT
TCAAGAGGTTATTCATTGATGAAGGGTTGATGTTGCATACTGGTTGTGTTAATTTTCTTGTGGCGATGTCATTGT
GCGAAATTGCATATGTTTACGGAGACACACAGCAGATTCCATACATCAATAGAGTTTCAGGATTCCCGTACCCCG
CCCATTTTGCCAAATTGGAAGTTGACGAGGTGGAGACACGCAGAACTACTCTCCGTTGTCCAGCCGATGTCACAC
ATTATCTGAACAGGAGATATGAGGGCTTTGTCATGAGCACTTCTTCGGTTAAAAAGTCTGTTTCGCAGGAGATGG
TCGGCGGAGCCGCCGTGATCAATCCGATCTCAAAACCCTTGCATGGCAAGATCCTGACTTTTACCCAATCGGATA
AAGAAGCTCTGCTTTCAAGAGGGTATTCAGATGTTCACACTGTGCATGAAGTGCAAGGCGAGACATACTCTGATG
TTTCACTAGTTAGGTTAACCCCTACACCGGTCTCCATCATTGCAGGAGACAGCCCACATGTTTTGGTCGCATTGT
CAAGGCACACCTGTTCGCTCAAGTACTACACTGTTGTTATGGATCCTTTAGTTAGTATCATTAGAGATCTAGAGA
AACTTAGCTCGTACTTGTTAGATATGTATAAGGTCGATGCAGGAACACAATAGCAATTACAGATTGACTCGGTGT
TCAAAGGTTCCAATCTTTTTGTTGCAGCGCCAAAGACTGGTGATATTTCTGATATGCAGTTTTACTATGATAAGT
GTCTCCCAGGCAACAGCACCATGATGAATAATTTTGATGCTGTTACCATGAGGTTGACTGACATTTCATTGAATG
TCAAAGATTGCATATTGGATATGTCTAAGTCTGTTGCTGCGCCTAAGGATCAAATCAAACCACTAATACCTATGG
TACGAACGGCGGCAGAAATGCCACGCCAGACTGGACTATTGGAAAATTTAGTGGCGATGATTAAAAGAAACTTTA

ACGCACCCGAGTTGTCTGGCATCATTGATATTGAAAATACTGCATCTTTGGTTGTAGATAAGTTTTTTGATAGTT
ATTTGCTTAAAGAAAAAAGAAAACCAAATAAAAATGTTTCTTTGTTCAGTAGAGAGTCTCTCAATAGATGGTTAG
AAAAGCAGGAACAGGTAACAATAGGCCAGCTCGCAGATTTTGATTTTGTGGATTTGCCAGCAGTTGATCAGTACA
GACACATGATTAAAGCACAACCCAAACAAAAGTTGGACACTTCAATCCAAACGGAGTACCCGGCTTTGCAGACGA
TTGTGTACCATTCAAAAAAGATCAATGCAATATTCGGCCCGTTGTTTAGTGAGCTTACTAGGCAATTACTGGACA
GTGTTGATTCGAGCAGATTTTTGTTTTTCACAAGAAAGACACCAGCGCAGATTGAGGATTTCTTCGGAGATCTCG
ACAGTCATGTGCCGATGGATGTCTTGGAGCTGGATATATCAAAATACGACAAATCTCAGAATGAATTCCACTGTG
CAGTAGAATACGAGATCTGGCGAAGATTGGGTTTTGAAGACTTCTTGGGAGAAGTTTGGAAACAAGGGCATAGAA
AGACCACCCTCAAGGATTATACCGCAGGTATAAAAACTTGCATCTGGTATCAAAGAAAGAGCGGGGACGTCACGA
CGTTCATTGGAAACACTGTGATCATTGCTGCATGTTTGGCCTCGATGCTTCCGATGGAGAAAATAATCAAAGGAG
CCTTTTGCGGTGACGATAGTCTGCTGTACTTTCCAAAGGGTTGTGAGTTTCCGGATGTGCAACACTCCGCAAATC
TTATGTGGAATTTTGAAGCAAAACTGTTTAAAAAACAGTATGGATACTTTTGCGGAAGATATGTAATACATCACG
ACAGAGGATGCATTGTGTATTACGATCCCCTAAAGTTGATCTCGAAACTTGGTGCTAAACACATCAAGGATTGGG
AACACTTGGAGGAGTTCAGAAGGTCTCTTTGTGATGTTGCTGTTTCGTTGAACAATTGTGCGTATTACACACAGT
TGGACGACGCTGTATGGGAGGTTCATAAGACCGCCCCTCCAGGTTCGTTTGTTTATAAAAGTCTGGTGAAGTATT
TGTCTGATAAAGTTCTTTTTAGAAGTTTGTTTATAGATGGCTCTAGTTGTTAAAGGAAAAGTGAATATCAATGAG
TTTATCGACCTGTCAAAAATGGAGAAGATCTTACCGTCGATGTTTACCCCTGTAAAGAGTGTTATGTGTTCCAAA
GTTGATAAAATAATGGTTCATGAGAATGAGTCATTGTCAGAGGTGAACCTTCTTAAAGGAGTTAAGCTTATTGAT
AGTGGATACGTCTGTTTAGCCGGTTTGGTCGTCACGGGCGAGTGGAACTTGCCTGACAATTGCAGAGGAGGTGTG
AGCGTGTGTCTGGTGGACAAAAGGATGGAAAGAGCCGACGAGGCCACTCTCGGATCTTACTACACAGCAGCTGCA
AAGAAAAGATTTCAGTTCAAGGTCGTTCCCAATTATGCTATAACCACCCAGGACGCGATGAAAAACGTCTGGCAA
GTTTTAGTTAATATTAGAAATGTGAAGATGTCAGCGGGTTTCTGTCCGCTTTCTCTGGAGTTTGTGTCGGTGTGT
ATTGTTTATAGAAATAATATAAAATTAGGTTTGAGAGAGAAGATTACAAACGTGAGAGACGGAGGGCCCATGGAA
CTTACAGAAGAAGTCGTTGATGAGTTCATGGAAGATGTCCCTATGTCGATCAGGCTTGCAAAGTTTCGATCTCGA
ACCGGAAAAAAGAGTGATGTCCGCAAAGGGAAAAATAGTAGTAGTGATCGGTCAGTGCCGAACAAGAACTATAGA
AATGTTAAGGATTTTGGAGGAATGAGTTTTAAAAAGAATAATTTAATCGATGATGATTCGGAGGCTACTGTCGCC
GAATCGGATTCGTTTTAAATATGTCTTACAGTATCACTACTCCATCTCAGTTCGTGTTCTTGTCATCAGCGTGGG
CCGACCCAATAGAGTTAATTAATTTATGTACTAATGCCTTAGGAAATCAGTTTCAAACACAACAAGCTCGAACTG
TCGTTCAAAGACAATTCAGTGAGGTGTGGAAACCTTCACCACAAGTAACTGTTAGGTTCCCTGACAGTGACTTTA
AGGTGTACAGGTACAATGCGGTATTAGACCCGCTAGTCACAGCACTGTTAGGTGCATTCGACACTAGAAATAGAA
TAATAGAAGTTGAAAATCAGGCGAACCCCACGACTGCCGAGACGTTAGATGCTACTCGTAGAGTAGACGACGCAA
CGGTGGCCATAAGGAGCGCGATAAATAATTTAATAGTAGAATTGATCAGAGGAACCGGATCTTATAATCGGAGCT
CTTTCGAGAGCTCTTCTGGTTTGGTTTGGACCTCTGGTCCTGCAACTTGAGGTAGTCAAGATGCATAATAAATAA
CGGATTGTGTCCGTAATCACACGTGGTGCGTACGATAACGCATAGTGTTTTTCCCTCCACTTAAATCGAAGGGTT
GTGTCTTGGATCGCGCGGGTCAAATGTATATGGTTCATATACATCCGCAGGCACGTAATAAAGCGAGGGGTTCGA
ATCCCCCCGTTACCCCCGGTAGGGGCCCAGGGTCGGCATGGCATCTCCACCTCCTCGCGGTCCGACCTGGGCTAC
TTCGGTAGGCTAAGGGAGAAGCGCTGAAATCACCAGTCTCTCTCTACAAATCTATCTCTCTCTATTTTCTCCATA
AATAATGTGTGAGTAGTTTCCCGATAAGGGAAATTAGGGTTCTTATAGGGTTTCGCTCATGTGTTGAGCATATAA
GAAACCCTTAGTATGTATTTGTATTTGTAAAATACTTCTATCAATAAAATTTCTAATTCCTAAAACCAAAATCCA
GGGGCCCTCGACGTTCCTTGACAGGATATATTGGCGGGTAAACTAAGTCGCTGTATGTGTTTGTTTGAGATCCTC
TAGGGCATGCAAGCTGATCTGGATCTCATGTGAGCAAAAGGCCAGCAAAAGGCCAGGAACCGTAAAAAGGCCGCG
TTGCTGGCGTTTTTCCATAGGCTCCGCCCCCCTGACGAGCATCACAAAAATCGACGCTCAAGTCAGAGGTGGCGA
AACCCGACAGGACTATAAAGATACCAGGCGTTTCCCCCTGGAAGCTCCCTCGTGCGCTCTCCTGTTCCGACCCTG
CCGCTTACCGGATACCTGTCCGCCTTTCTCCCTTCGGGAAGCGTGGCGCTTTCTCATAGCTCACGCTGTAGGTAT
CTCAGTTCGGTGTAGGTCGTTCGCTCCAAGCTGGGCTGTGTGCACGAACCCCCCGTTCAGCCCGACCGCTGCGCC
TTATCCGGTAACTATCGTCTTGAGTCCAACCCGGTAAGACACGACTTATCGCCACTGGCAGCAGCCACTGGTAAC
AGGATTAGCAGAGCGAGGTATGTAGGCGGTGCTACAGAGTTCTTGAAGTGGTGGCCTAACTACGGCTACACTAGA
AGAACAGTATTTGGTATCTGCGCTCTGCTGAAGCCAGTTACCTTCGGAAGAAGAGTTGGTAGCTCTTGATCCGGC
AAACAAACCACCGCTGGTAGCGGTGGTTTTTTTGTTTGCAAGCAGCAGATTACGCGCAGAAAAAAAGGATCTCAA
GAAGATCCTTTGATCTTTTCTACGGGGTCTGACGCTCAGTGGAACGAAAACTCACGTTAAGGGATTTTGGTCATG
AGATTATCAAAAAGGATCTTCACCTAGATCCTTTTAAATTAAAAATGAAGTTTTAAATCAATCTAAAGTATATAT
GTGTAACATTGGTCTAGTGATTAGAAAAACTCATCGAGCATCAAATGAAACTGCAATTTATTCATATCAGGATTA
TCAATACCATATTTTTGAAAAAGCCGTTTCTGTAATGAAGGAGAAAACTCACCGAGGCAGTTCCATAGGATGGCA
AGATCCTGGTATCGGTCTGCGATTCCGACTCGTCCAACATCAATACAACCTATTAATTTCCCCTCGTCAAAAATA
AGGTTATCAAGTGAGAAATCACCATGAGTGACGACTGAATCCGGTGAGAATGGCAAAAGTTTATGCATTTCTTTC
CAGACTTGTTCAACAGGCCAGCCATTACGCTCGTCATCAAAATCACTCGCATCAACCAAACCGTTATTCATTCGT
GATTGCGCCTGAGCAAGACGAAATACGCGATCGCTGTTAAAAGGACAATTACAAACAGGAATCGAATGCAACCGG
CGCAGGAACACTGCCAGCGCATCAACAATATTTTCACCTGAATCAGGATATTCTTCTAATACCTGGAATGCTGTT
TTCCCTGGGATCGCAGTGGTGAGTAACCATGCATCATCAGGAGTACGGATAAAATGCTTGATGGTCGGAAGAGGC
ATAAATTCCGTCAGCCAGTTTAGTCTGACCATCTCATCTGTAACAACATTGGCAACGCTACCTTTGCCATGTTTC
AGAAACAACTCTGGCGCATCGGGCTTCCCATACAATCGGTAGATTGTCGCACCTGATTGCCCGACATTATCGCGA

GCCCATTTATACCCATATAAATCAGCATCCATGTTGGAATTTAATCGCGGCCTTGAGCAAGACGTTTCCCGTTGA
ATATGGCTCATAACACCCCTTGTATTACTGTTTATGTAAGCAGACAGTTTTATTGTTCATGATGATATATTTTTA
TCTTGTGCAATGTAACATCAGAGATTTTGAGACACAACGTGGCTTTGTTGAATAAATCGAACTTTTGCTGAGTTG
AAGGATCAGATCACGCATCTTCCCGACAACGCAGACCGTTCCGTGGCAAAGCAAAAGTTCAAAATCACCAACTGG
TCCACCTACAACAAAGCTCTCATCAACCGTGGCTCCCTCACTTTCTGGCTGGATGATGGGGCGATTCAGGCGATC
CCCATCCAACAGCCCGCCGTCGAGCGGGCTTTTTTATCCCCGGAAGCCTGTGGATAGAGGGTAGTTATCCACGTG
AAACCGCTAATGCCCCGCAAAGCCTTGATTCACGGGGCTTTCCGGCCCGCTCCAAAAACTATCCACGTGAAATCG
CTAATCAGGGTACGTGAAATCGCTAATCGGAGTACGTGAAATCGCTAATAAGGTCACGTGAAATCGCTAATCAAA
AAGGCACGTGAGAACGCTAATAGCCCTTTCAGATCAACAGCTTGCAAACACCCCTCGCTCCGGCAAGTAGTTACA
GCAAGTAGTATGTTCAATTAGCTTTTCAATTATGAATATATATATCAATTATTGGTCGCCCTTGGCTTGTGGACA
ATGCGCTACGCGCACCGGCTCCGCCCGTGGACAACCGCAAGCGGTTGCCCACCGTCGAGCGCCTTTGCCCACAAC
CCGGCGGCCGGCCGCAACAGATCGTTTTATAAATTTTTTTTTGAAAAAGAAAAAGCCCGAAAGGCGGCAACCT
CTCGGGCTTCTGGATTTCCGATCCCCGGAATTAGATCCGTTTAAACTACGTAAGATCGATCTTGGCAGGATATAT
TGTGGTGTAAACGTTCCTGCGGCGGTCGAGATGGATCTTGGCAGGATATATTGTGGTGTAAACGTTCCT

TMV-MK087763 is in bold. ***Cauliflower mosaic virus* (CaMV) 35S promoter** is in red with the transcription +1 nucleotide on yellow background. **Hepatitis delta virus (HDV)-derived ribozyme** is in red. **CaMV 35S terminator** is in fuchsia with the processing and polyadenylation site underlined. *E. coli* pUC replication origin is in blue on gray background. *Kanamycin* selection marker is in blue on dark gray background (complementary strand). *A. tumefaciens* pSa replication origin is in blue on gray background. *A. tumefaciens* T-DNA **RB** with **overdrive** (underlined) is in blue on yellow background and **double LB** is in blue on red background.

>pMTMVi-N (2376 bp)
AAGATTACAAACGTGAGAGACGGAGGGCCCATGGAACTTACAGAAGAAGTCGTTGATGAGTTCATGGAAGATGTC
CCTATGTCGATCAGGCTTGCAAAGTTTCGATCTCGAACCGGAAAAAAGAGTGATGTCCGCAAAGGGAAAAATAGT
AGTAGTGATCGGTCAGTGCCGAACAAGAACTATAGAAATGTTAAGGATTTTGGAGGAATGAGTTTTAAAAAGAAT
AATTTAATCGATGATGATTCGGAGGCTACTGTCGCCGAATCGGATTCGTTTTAAATAGATCTTACAGTATCACTA
CTCCATCTCAGTTCGTGTTCTTGTCACCGGTGAAAGCGGGCAGTGAGCGCAACGCAATTAATGTGAGTTAGCTCA
CTCATTAGGCACCCCAGGCTTTACACTTTATGCTCCCGGCTCGTATGTTGTGTGGAATTGTGAGCGGATAACAAT
TTCACACAGGAAACAGCTATGACCATGATTACGCCAAGCGCGCAATTAACCCTCACTAAAGGGAACAAAAGCTGG
GTACCCAATTCGCCCTATAGTGAGTCGTATTACGCGCGCTCACTGGCCGTCGTTTTACAACGTCGTGACTGGGAA
AACCCTGGCGTTACCCAACTTAATCGCCTTGCAGCACATCCCCCTTTCGCCAGCTGGCGTAATAGCGAAGAGGCC
CGCACCGATCGCCCTTCCCAACAGTTGCGCAGCCTGAATGGCGAATGGACGCGCTCGAGGTCCTGCAACTTGAG
GTAGTCAAGATGCATAATAAATAACGGATTGTGTCCGTAATCACACGTGGTGCGTACGATAACGCATAGTGTTTT
TCCCTCCACTTGGAGACCGCGTTGCTGGCGTTTTCCATAGGCTCCGCCCCCCTGACGAGCATCACAAAAATCGA
CGCTCAAGTCAGAGGTGGCGAAACCCGACAGGACTATAAAGATACCAGGCGTTTCCCCCTGGAAGCTCCCTCGTG
CGCTCTCCTGTTCCGACCCTGCCGCTTACCGGATACCTGTCCGCCTTTCTCCCTTCGGGAAGCGTGGCGCTTTCT
CATAGCTCACGCTGTAGGTATCTCAGTTCGGTGTAGGTCGTTCGCTCCAAGCTGGGCTGTGTGCACGAACCCCCC
GTTCAGCCCGACCGCTGCGCCTTATCCGGTAACTATCGTCTTGAGTCCAACCCGGTAAGACACGACTTATCGCCA
CTGGCAGCAGCCACTGGTAACAGGATTAGCAGAGCGAGGTATGTAGGCGGTGCTACAGAGTTCTTGAAGTGGTGG
CCTAACTACGGCTACACTAGAAGGACAGTATTTGGTATCTGCGCTCTGCTGAAGCCAGTTACCTTCGGAAAAAGA
GTTGGTAGCTCTTGATCCGGCAAACAAACCACCGCTGGTAGCGGTGGTTTTTTTGTTTGCAAGCAGCAGATTACG
CGCAGAAAAAAAGGATCTCAAGAAGATCCTTTTTACCAATGCTTAATCAGTGAGGCACCTATCTCAGCGATCTGT
CTATTTCGTTCATCCATAGTTGCCTGACTCCCCGTCGTGTAGATAACTACGATACGGGAGGGCTTACCATCTGGC
CCCAGTGCTGCAATGATACCGCGAGAGCCACGCTCACCGGCTCCAGATTTATCAGCAATAAACCAGCCAGCCGGA
AGGGCCGAGCGCAGAAGTGGTCCTGCAACTTTATCCGCCTCCATCCAGTCTATTAATTGTTGCCGGGAAGCTAGA
GTAAGTAGTTCGCCAGTTAATAGTTTGCGCAACGTTGTTGCCATTGCTACAGGCATCGTGGTGTCACGCTCGTCG
TTTGGTATGGCTTCATTCAGCTCCGGTTCCCAACGATCAAGGCGAGTTACATGATCCCCCATGTTGTGCAAAAAA
GCGGTTAGCTCCTTCGGTCCTCCGATCGTTGTCAGAAGTAAGTTGGCCGCAGTGTTATCACTCATGGTTATGGCA
GCACTGCATAATTCTCTTACTGTCATGCCATCCGTAAGATGCTTTTCTGTGACTGGTGAGTACTCAACCAAGTCA
TTCTGAGAATAGTGTATGCGGCGACCGAGTTGCTCTTGCCCGGCGTCAATACGGGATAATACCGCGCCACATAGC
AGAACTTTAAAAGTGCTCATCATTGGAAAACGTTCTTCGGGGCGAAAACTCTCAAGGATCTTACCGCTGTTGAGA
TCCAGTTCGATGTAACCCACTCGTGCACCCAACTGATCTTCAGCATCTTTTACTTTCACCAGCGTTTCTGGGTGA
GCAAAAACAGGAAGGCAAAATGCCGCAAAAAAGGGAATAAGGGCGACACGGAAATGTTGAATACTCATACTCTTC
CTTTTTCAATATTATTGAAGCATTTATCAGGGTTATTGTCTCATGGTCTCG

TMV-MK087763 genome fragment is in bold. The **ATG** to **AGA** mutation of the TMV CP initiation codon is indicated. Unique restriction sites ***Nco*I** and *Pfl*23II are on green background. A polylinker is inserted between TMV positions 5756 and 6177. The polylinker consists of two unique restriction sites ***Age*I** and ***Xho*I** (yellow background) separated by a blue-white *LacZ* marker (in blue and italics). Two ***Bsa*I** recognition sites to release the TMV fragment, once manipulated, are indicated on blue background. Hybridization sites for PI and PII oligonucleotides to alternatively produce the manipulated TMV fragment by PCR are doubly underlined. Plasmid also contains pUC replication origin (in gray) and ampicillin resistance marker (on gray background).

```
>pMTMVi-M (3373 bp)
AAGATTACAAACGTGAGAGACGGAGGGCCCATGGAACTTACAGAAGAAGTCGTTGATGAGTTCATGGAAGATGTC
CCTATGTCGATCAGGCTTGCAAAGTTTCGATCTCGAACCGGAAAAAAGAGTGATGTCCGCAAAGGGAAAAATAGT
AGTAGTGATCGGTCAGTGCCGAACAAGAACTATAGAAATGTTAAGGATTTTGGAGGAATGAGTTTTAAAAAGAAT
AATTTAATCGATGATGATTCGGAGGCTACTGTCGCCGAATCGGATTCGTTTTAAATAGATCTTACAGTATCACTA
CTCCATCTCAGTTCGTGTTCTTGTCACCGGTGAAAGCGGGCAGTGAGCGCAACGCAATTAATGTGAGTTAGCTCA
CTCATTAGGCACCCCAGGCTTTACACTTTATGCTCCCGGCTCGTATGTTGTGTGGAATTGTGAGCGGATAACAAT
TTCACACAGGAAACAGCTATGACCATGATTACGCCAAGCGCGCAATTAACCCTCACTAAAGGGAACAAAAGCTGG
GTACCCAATTCGCCCTATAGTGAGTCGTATTACGCGCGCTCACTGGCCGTCGTTTTACAACGTCGTGACTGGGAA
AACCCTGGCGTTACCCAACTTAATCGCCTTGCAGCACATCCCCCTTTCGCCAGCTGGCGTAATAGCGAAGAGGCC
CGCACCGATCGCCCTTCCCAACAGTTGCGCAGCCTGAATGGCGAATGGGACGCGCTCGAGGTCCTGCAACTTGAG
GTAGTCAAGATGCATAATAAATAACGGATTGTGTCCGTAATCACACGTGGTGCGTACGATAACGCATAGTGTTTT
TCCCTCCACTTAAATCGAAGGGTTGTGTCTTGGATCGCGCGGTCAAATGTATATGGTTCATATACATCCGCAGG
CACGTAATAAAGCGAGGGGTTCGGAACCAAATCCTCAAAAAGAGGTCCGAAAAATAATAATAATTTAGGTAAGGG
GCGTTCAGGCGGAAGGCCTAAACCAAAAAGTTTTGATGAAGTTGAAAAAGAGTTTGATAATTTGATTGAAGATGA
AGCCGAGACGTCGGTCGCGGATTCTGATTCGTATTAAATATGTCTTACTCAATCACTTCTCCATCGCAATTTGTG
TTTTTGTCATCTGTATGGGCTGACCCTATAGAATTGTTAAACGTTTGTACAAATTCGTTAGGTAACCAGTTTCAA
ACACAGCAAGCAAGAACTACTGTTCAACAGCAGTTCAGCGAGGTGTGGAAACCTTTCCCTCAGAGCACCGTCAGA
TTTCCTGGCGATGTTTATAAGGTGTACAGGTACAATGCAGTTTTAGATCCTCTAATTACTGCGTTGCTGGGGTCT
TTCGATACTAGGAATAGAATAATCGAAGTAGAAAACCAGCAGAATCCGACAACAGCTGAAACGTTAGATGCTACC
CGCAGGGTAGACGACGCTACGGTTGCAATTCGGTCTGCTATAAATAATTTAGTTAATGAACTAGTAAGAGGTACT
GGACTGTACAATCAAAATACTTTTGAAAGTATGTCTGGGTTGGTCTGGACCTCTGCACCTGCATCTTAAATGCAT
AGGTGCTGAAATATAAAGTTTGTGTTTCTAAAACACACGTGGTACGTACGATAACGTATAGTGTTTTTCCCTCCA
CTTAAATCGAAAGGGTAGTGTCTTGGAGCGCGCGGAGTAAACATATATGGTTCATATATGTCCGTAGCACGTAAA
AAAGCGAGGGATTCGAATTCCCCCGGAACCCCCGGGTTGGGGCCCAGGGTCGGCATGGCATCTCCACCTCCTCGC
GGTCCGACCTGGGCTACTTCGGTAGGCTAAGGGGGAGACCGCGTTGCTGGCGTTTTTCCATAGGCTCCGCCCCCC
TGACGAGCATCACAAAAATCGACGCTCAAGTCAGAGGTGGCGAAACCCGACAGGACTATAAAGATACCAGGCGTT
TCCCCCTGGAAGCTCCCTCGTGCGCTCTCCTGTTCCGACCCTGCCGCTTACCGGATACCTGTCCGCCTTTCTCCC
TTCGGGAAGCGTGGCGCTTTCTCATAGCTCACGCTGTAGGTATCTCAGTTCGGTGTAGGTCGTTCGCTCCAAGCT
GGGCTGTGTGCACGAACCCCCCGTTCAGCCCGACCGCTGCGCCTTATCCGGTAACTATCGTCTTGAGTCCAACCC
GGTAAGACACGACTTATCGCCACTGGCAGCAGCCACTGGTAACAGGATTAGCAGAGCGAGGTATGTAGGCGGTGC
TACAGAGTTCTTGAAGTGGTGGCCTAACTACGGCTACACTAGAAGGACAGTATTTGGTATCTGCGCTCTGCTGAA
GCCAGTTACCTTCGGAAAAAGAGTTGGTAGCTCTTGATCCGGCAAACAAACCACCGCTGGTAGCGGTGGTTTTTT
TGTTTGCAAGCAGCAGATTACGCGCAGAAAAAAAGGATCTCAAGAAGATCCTTTTTACCAATGCTTAATCAGTGA
GGCACCTATCTCAGCGATCTGTCTATTTCGTTCATCCATAGTTGCCTGACTCCCCGTCGTGTAGATAACTACGAT
ACGGGAGGGCTTACCATCTGGCCCCAGTGCTGCAATGATACCGCGAGAGCCACGCTCACCGGCTCCAGATTTATC
AGCAATAAACCAGCCAGCCGGAAGGGCCGAGCGCAGAAGTGGTCCTGCAACTTTATCCGCCTCCATCCAGTCTAT
TAATTGTTGCCGGGAAGCTAGAGTAAGTAGTTCGCCAGTTAATAGTTTGCGCAACGTTGTTGCCATTGCTACAGG
CATCGTGGTGTCACGCTCGTCGTTTGGTATGGCTTCATTCAGCTCCGGTTCCCAACGATCAAGGCGAGTTACATG
ATCCCCCATGTTGTGCAAAAAAGCGGTTAGCTCCTTCGGTCCTCCGATCGTTGTCAGAAGTAAGTTGGCCGCAGT
GTTATCACTCATGGTTATGGCAGCACTGCATAATTCTCTTACTGTCATGCCATCCGTAAGATGCTTTTCTGTGAC
TGGTGAGTACTCAACCAAGTCATTCTGAGAATAGTGTATGCGGCGACCGAGTTGCTCTTGCCCGGCGTCAATACG
GGATAATACCGCGCCACATAGCAGAACTTTAAAAGTGCTCATCATTGGAAAACGTTCTTCGGGGCGAAAACTCTC
AAGGATCTTACCGCTGTTGAGATCCAGTTCGATGTAACCCACTCGTGCACCCAACTGATCTTCAGCATCTTTTAC
TTTCACCAGCGTTTCTGGGTGAGCAAAAACAGGAAGGCAAAATGCCGCAAAAAAGGGAATAAGGGCGACACGGAA
ATGTTGAATACTCATACTCTTCCTTTTTCAATATTATTGAAGCATTTATCAGGGTTATTGTCTCATGGTCTCG
```

TMV-MK087763 genome fragment is in bold. The **ATG** to **AGA** mutation of the TMV CP initiation codon is indicated. Unique restriction sites ***Nco*I**, *Pfl*23II and ***Cpo*I** are on green

background. A polylinker is inserted between TMV positions 5756 and 6177. The polylinker consists of two unique restriction sites ***Age*I** and ***Xho*I** (yellow background) separated by a blue-white *LacZ* marker (in blue and italics). 3' fragment of tomato mosaic virus (ToMV) genome (CP promoter, CP ORF and 3' UTR) is on gray background (**transcription initiation** and **start** and **stop** codons are indicated). **HDV-derived ribozyme** is in red. Two ***Bsa*I** recognition sites to release the manipulated TMV fragment are indicated on blue background. Hybridization sites for PI and PIII oligonucleotides to alternatively produce the manipulated TMV fragment by PCR are doubly underlined. Plasmid also contains pUC replication origin (in gray) and ampicillin resistance marker (on gray background).

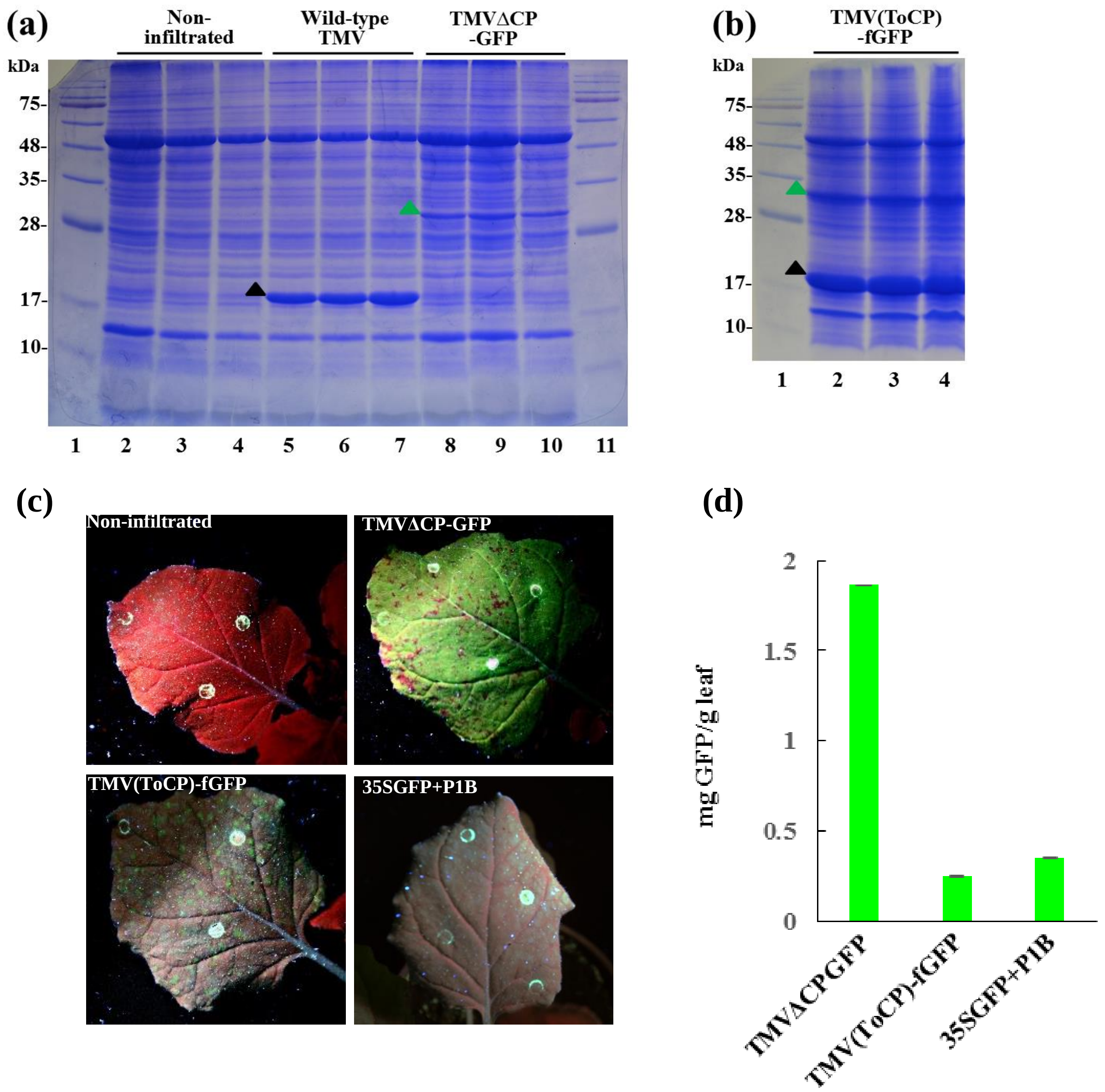

Figure S3. Analysis of GFP produced in *N. benthamiana* plants using the TMV vector system. Total proteins from plant leaves were separated by SDS-PAGE in 12.5% polyacrylamide gels and stained with Coomassie blue. (a) Lanes 1 and 11, and (b) lane 1, marker proteins with the sizes in kDa on the left. (a) Proteins from tissues of three independent plants non-infiltrated (lanes 2 to 4), or infiltrated with wild-type TMV (lanes 5 to 7) or TMVΔCP-GFP (lanes 8 to 10). Tissues were collected 7 dpi. (b) Proteins from upper non-inoculated tissues from three independent plants agroinoculated with TMV(ToCP)-fGFP (lanes 2 to 4). Tissues were collected 13 dpi. Green and black arrowheads points to GFP and TMV CP. (c) Appearance of *N. benthamiana* leaves agroinfiltrated with the indicated TMV vectors, or the binary vector pGWB5 along with the silencing suppressor P1b from *Cucumber vein yellowing virus* (CMYV) at 7 dpi under UV light. (d) Comparative analysis of GFP production in the agroinfiltrated leaves with the two viral vectors and a simple 35S promoter using the P1b silencing suppressor at 7 dpi.

Figure S4. Sequence of TMV-derived recombinant viruses TMVΔCP-GFP, TMVΔCP-AfpB, TMVΔCP-AfpBKDEL, TMVΔCP-UP, TMVΔCP-UPKDEL, TMVΔCP-UPVS, TMVΔCP-AgAFP and TMV(ToCP)-fGFP.

>TMV (with the ATG to AGA mutation of CP start codon)

```
GTATTTTTACAACAATTACCAACAACAACAAACAACAAACAACATTACAATTACTATTTACAATTACAATGGCAT
ACACACAGACAGCTACCACATCAGCTTTGCTGGACACTGTCCGAGGAAACAACTCCTTGGTCAATGATCTAGCAA
AGCGTCGTCTTTACGACACAGCGGTTGAAGAGTTTAACGCTCGTGACCGCAGGCCCAAGGTGAACTTTTCAAAAG
TAATAAGCGAGGAGCAGACGCTTATTGCTACCCGGGCGTATCCAGAATTCCAAATTACATTTTATAACACGCAAA
ATGCCGTGCATTCGCTTGCAGGTGGATTGCGATCTTTAGAACTGGAATATCTGATGATGCAAATTCCCTACGGAT
CATTGACTTATGACATAGGCGGGAATTTTGCATCGCATCTGTTCAAGGGACGAGCATATGTACACTGCTGCATGC
CCAACCTGGACGTTCGAGACATCATGCGGCACGAAGGCCAGAAAGACAGTATTGAACTATACCTTTCTAGGCTAG
AGAGAGGGGGGAAAACAGTCCCCAACTTCCAAAAGGAAGCATTTGACAGATACGCAGAAATTCCTGAAGACGCTG
TCTGTCACAATACTTTCCAGACATGCGAACATCAGCCGATGCAGCAATCAGGCAGAGTGTATGCCATTGCGCTAC
ACAGCATATATGACATACCAGCCGATGAGTTCGGGGCGGCACTCTTGAGGAAAAATGTCCATACGTGCTATGCCG
CTTTCCACTTCTCCGAGAACCTGCTTCTTGAAGATTCATGCGTCAATTTGGACGAAATCAACGCGTGTTTTTCGC
GCGATGGAGACAAGTTGACCTTTTCTTTTGCATCAGAGAGTACTCTTAATTACTGTCATAGTTATTCTAATATTC
TTAAGTATGTGTGCAAAACTTACTTCCCGGCCTCTAATAGAGAGGTTTACATGAAGGAGTTTTTAGTCACCAGAG
TTAATACCTGGTTTTGTAAGTTTTCTAGAATAGATACTTTTCTTTTGTACAAAGGTGTGGCCCATAAAAGTGTAG
ATAGTGAGCAGTTTTATACTGCAATGGAAGACGCATGGCATTACAAAAAGACTCTTGCAATGTGCAACAGCGAGA
GAATCCTCCTTGAGGATTCATCATCAGTCAATTACTGGTTTCCCAAAATGAGGGATATGGTCATCGTACCATTAT
TCGACATTTCTTTGGAGACTAGTAAGAGGACGCGCAAGGAAGTCTTAGTGTCCAAGGATTTCGTGTTTACAGTGC
TTAACCACATTCGAACATACCAGGCGAAAGCTCTTACATACGCAAATGTTTTGTCCTTCGTCGAATCGATTCGAT
CGAGGGTAATCATTAACGGTGTGACAGCGAGGTCCGAATGGGATGTGGACAAATCTTTGTTACAATCCTTGTCCA
TGACGTTTTACCTGCATACTAAGCTTGCCGTTCTAAAGGATGACTTACTGATTAGCAAGTTTAGTCTCGGTTCGA
AAACGGTGTGCCAGCATGTGTGGGATGAGATTTCGCTGGCGTTTGGGAACGCATTTCCCTCCGTGAAAGAGAGGC
TCTTGAACAGGAAACTTATCAGAGTGGCAGGCGACGCATTAGAGATCAGGGTGCCTGATCTATATGTGACCTTCC
ACGACAGATTAGTGACTGAGTACAAGGCCTCTGTGGACATGCCTGCGCTTGACATTAGGAAGAAGATGGAAGAAA
CGGAAGTGATGTACAATGCACTTTCAGAATTATCGGTGTTAAGGGAGTCTGACAAATTCGATGTTGATGTTTTTT
CCCAGATGTGCCAATCTTTGGAAGTTGACCCAATGACGGCAGCGAAGGTTATAGTCGCGGTCATGAGCAATGAGA
GCGGTCTGACTCTCACATTTGAACGACCTACTGAGGCGAATGTTGCGCTAGCTTTACAGGATCAAGAGAAGGCTT
CAGAAGGTGCATTGGTAGTTACCTCAAGAGAAGTTGAAGAACCGTCCATGAAGGGTTCGATGGCCAGAGGAGAGT
TACAATTAGCTGGTCTTGCTGGAGATCATCCGGAGTCGTCCTATTCTAAGAACGAGGAGATAGAGTCTTTAGAGC
AGTTTCATATGGCGACGGCAGATTCGTTAATTCGTAAGCAGATGAGCTCGATTGTGTACACGGGTCCGATTAAAG
TTCAGCAAATGAAAAACTTTATCGATAGCCTGGTAGCATCACTATCTGCTGCGGTGTCGAATCTCGTCAAGATCC
TCAAAGATACAGCTGCTATTGACCTTGAAACCCGTCAAAAGTTTGGAGTCTTGGATGTTGCATCTAGGAAGTGGT
TAATCAAACCAACGGCCAAGAGTCATGCATGGGGTGTTGTTGAAACCCACGCGAGGAAGTATCATGTGGCGCTTT
TGGAATATGATGAGCAGGGTGTGGTGACATGCGATGATTGGAGAAGAGTAGCTGTTAGCTCTGAGTCTGTTGTTT
ATTCCGACATGGCGAAACTCAGAACTCTGCGCAGACTGCTTCGAAACGGAGAACCGCATGTCAGTAGCGCAAAGG
TTGTTCTTGTGGACGGAGTTCCGGGCTGTGGAAAAACCAAAGAAATTCTTTCCAGGGTTAATTTTGATGAAGATC
TAATTTTAGTACCTGGGAAGCAAGCCGCGGAAATGATCAGAAGACGTGCGAATTCCTCAGGGATTATTGTGGCCA
CGAAGGACAACGTTAAAACCGTTGATTCTTTCATGATGAATTTTGGGAAAAGCACACGCTGTCAGTTCAAGAGGT
TATTCATTGATGAAGGGTTGATGTTGCATACTGGTTGTGTTAATTTTCTTGTGGCGATGTCATTGTGCGAAATTG
CATATGTTTACGGAGACACACAGCAGATTCCATACATCAATAGAGTTTCAGGATTCCCGTACCCCGCCCATTTTG
CCAAATTGGAAGTTGACGAGGTGGAGACACGCAGAACTACTCTCCGTTGTCCAGCCGATGTCACACATTATCTGA
ACAGGAGATATGAGGGCTTTGTCATGAGCACTTCTTCGGTTAAAAAGTCTGTTTCGCAGGAGATGGTCGGCGGAG
CCGCCGTGATCAATCCGATCTCAAAACCCTTGCATGGCAAGATCCTGACTTTTACCCAATCGGATAAAGAAGCTC
TGCTTTCAAGAGGGTATTCAGATGTTCACACTGTGCATGAAGTGCAAGGCGAGACATACTCTGATGTTTCACTAG
TTAGGTTAACCCCTACACCGGTCTCCATCATTGCAGGAGACAGCCCACATGTTTTGGTCGCATTGTCAAGGCACA
CCTGTTCGCTCAAGTACTACACTGTTGTTATGGATCCTTTAGTTAGTATCATTAGAGATCTAGAGAAACTTAGCT
CGTACTTGTTAGATATGTATAAGGTCGATGCAGGAACACAATAGCAATTACAGATTGACTCGGTGTTCAAAGGTT
CCAATCTTTTTGTTGCAGCGCCAAAGACTGGTGATATTTCTGATATGCAGTTTTACTATGATAAGTGTCTCCCAG
GCAACAGCACCATGATGAATAATTTTGATGCTGTTACCATGAGGTTGACTGACATTTCATTGAATGTCAAAGATT
GCATATTGGATATGTCTAAGTCTGTTGCTGCGCCTAAGGATCAAATCAAACCACTAATACCTATGGTACGAACGG
CGGCAGAAATGCCACGCCAGACTGGACTATTGGAAAATTTAGTGGCGATGATTAAAAGAAACTTTAACGCACCCG
AGTTGTCTGGCATCATTGATATTGAAAATACTGCATCTTTGGTTGTAGATAAGTTTTTTGATAGTTATTTGCTTA
AAGAAAAAAGAAAACCAAATAAAAATGTTTCTTTGTTCAGTAGAGAGTCTCTCAATAGATGGTTAGAAAAGCAGG
AACAGGTAACAATAGGCCAGCTCGCAGATTTTGATTTTGTGGATTTGCCAGCAGTTGATCAGTACAGACACATGA
TTAAAGCACAACCCAAACAAAAGTTGGACACTTCAATCCAAACGGAGTACCCGGCTTTGCAGACGATTGTGTACC
ATTCAAAAAAGATCAATGCAATATTCGGCCCGTTGTTTAGTGAGCTTACTAGGCAATTACTGGACAGTGTTGATT
```

CGAGCAGATTTTTGTTTTTCACAAGAAAGACACCAGCGCAGATTGAGGATTTCTTCGGAGATCTCGACAGTCATG
TGCCGATGGATGTCTTGGAGCTGGATATATCAAAATACGACAAATCTCAGAATGAATTCCACTGTGCAGTAGAAT
ACGAGATCTGGCGAAGATTGGGTTTTGAAGACTTCTTGGGAGAAGTTTGGAAACAAGGGCATAGAAAGACCACCC
TCAAGGATTATACCGCAGGTATAAAAACTTGCATCTGGTATCAAAGAAAGAGCGGGGACGTCACGACGTTCATTG
GAAACACTGTGATCATTGCTGCATGTTTGGCCTCGATGCTTCCGATGGAGAAAATAATCAAAGGAGCCTTTTGCG
GTGACGATAGTCTGCTGTACTTTCCAAAGGGTTGTGAGTTTCCGGATGTGCAACACTCCGCAAATCTTATGTGGA
ATTTTGAAGCAAAACTGTTTAAAAAACAGTATGGATACTTTTGCGGAAGATATGTAATACATCACGACAGAGGAT
GCATTGTGTATTACGATCCCCTAAAGTTGATCTCGAAACTTGGTGCTAAACACATCAAGGATTGGGAACACTTGG
AGGAGTTCAGAAGGTCTCTTTGTGATGTTGCTGTTTCGTTGAACAATTGTGCGTATTACACACAGTTGGACGACG
CTGTATGGGAGGTTCATAAGACCGCCCCTCCAGGTTCGTTTGTTTATAAAAGTCTGGTGAAGTATTTGTCTGATA
AAGTTCTTTTTAGAAGTTTGTTTATAGATGGCTCTAGTTGTTAAAGGAAAAGTGAATATCAATGAGTTTATCGAC
CTGTCAAAAATGGAGAAGATCTTACCGTCGATGTTTACCCCTGTAAAGAGTGTTATGTGTTCCAAAGTTGATAAA
ATAATGGTTCATGAGAATGAGTCATTGTCAGAGGTGAACCTTCTTAAAGGAGTTAAGCTTATTGATAGTGGATAC
GTCTGTTTAGCCGGTTTGGTCGTCACGGGCGAGTGGAACTTGCCTGACAATTGCAGAGGAGGTGTGAGCGTGTGT
CTGGTGGACAAAAGGATGGAAAGAGCCGACGAGGCCACTCTCGGATCTTACTACACAGCAGCTGCAAAGAAAAGA
TTTCAGTTCAAGGTCGTTCCCAATTATGCTATAACCACCCAGGACGCGATGAAAAACGTCTGGCAAGTTTTAGTT
AATATTAGAAATGTGAAGATGTCAGCGGGTTTCTGTCCGCTTTCTCTGGAGTTTGTGTCGGTGTGTATTGTTTAT
AGAAATAATATAAAATTAGGTTTGAGAGAGAAGATTACAAACGTGAGAGACGGAGGGCCCATGGAACTTACAGAA
GAAGTCGTTGATGAGTTCATGGAAGATGTCCCTATGTCGATCAGGCTTGCAAAGTTTCGATCTCGAACCGGAAAA
AAGAGTGATGTCCGCAAAGGGAAAAATAGTAGTAGTGATCGGTCAGTGCCGAACAAGAACTATAGAAATGTTAAG
GATTTTGGAGGAATGAGTTTTAAAAAGAATAATTTAATCGATGATGATTCGGAGGCTACTGTCGCCGAATCGGAT
TCGTTTTAAAT**AGA**TCTTACAGTATCACTACTCCATCTCAGTTCGTGTTCTTGTCA*TCAGCGTGGGCCGACCCAA*
TAGAGTTAATTAATTTATGTACTAATGCCTTAGGAAATCAGTTTCAAACACAACAAGCTCGAACTGTCGTTCAAA
GACAATTCAGTGAGGTGTGGAAACCTTCACCACAAGTAACTGTTAGGTTCCCTGACAGTGACTTTAAGGTGTACA
GGTACAATGCGGTATTAGACCCGCTAGTCACAGCACTGTTAGGTGCATTCGACACTAGAAATAGAATAATAGAAG
TTGAAAATCAGGCGAACCCCACGACTGCCGAGACGTTAGATGCTACTCGTAGAGTAGACGACGCAACGGTGGCCA
TAAGGAGCGCGATAAATAATTTAATAGTAGAATTGATCAGAGGAACCGGATCTTATAATCGGAGCTCTTTCGAGA
*GCTCTTCTGGTTTGGTTTGGACCTCT*GGTCCTGCAACTTGAGGTAGTCAAGATGCATAATAAATAACGGATTGTG
TCCGTAATCACACGTGGTGCGTACGATAACGCATAGTGTTTTTCCCTCCACTTAAATCGAAGGGTTGTGTCTTGG
ATCGCGCGGGTCAAATGTATATGGTTCATATACATCCGCAGGCACGTAATAAAGCGAGGGGTTCGAATCCCCCCG
TTACCCCCGGTAGGGGCCCA

>TMVΔCP-GFP (sequence that replaces nucleotides 5757 to 6176 in TMV)
ATGGTGAGCAAGGGCGAGGAGCTGTTCACCGGGGTGGTGCCCATCCTGGTCGAGCTGGACGGCGACGTAAACGGC
CACAAGTTCAGCGTGTCCGGCGAGGGCGAGGGCGATGCCACCTACGGCAAGCTGACCCTGAAGTTCATCTGCACC
ACCGGCAAGCTGCCCGTGCCCTGGCCCACCCTCGTGACCACCCTGACCTACGGCGTGCAGTGCTTCAGCCGCTAC
CCCGACCACATGAAGCAGCACGACTTCTTCAAGTCCGCCATGCCCGAAGGCTACGTCCAGGAGCGCACCATCTTC
TTCAAGGACGACGGCAACTACAAGACCCGCGCCGAGGTGAAGTTCGAGGGCGACACCCTGGTGAACCGCATCGAG
CTGAAGGGCATCGACTTCAAGGAGGACGGCAACATCCTGGGGCACAAGCTGGAGTACAACTACAACAGCCACAAC
GTCTATATCATGGCCGACAAGCAGAAGAACGGCATCAAGGTGAACTTCAAGATCCGCCACAACATCGAGGACGGC
AGCGTGCAGCTCGCCGACCACTACCAGCAGAACACCCCCATCGGCGACGGCCCCGTGCTGCTGCCCGACAACCAC
TACCTGAGCACCCAGTCCGCCCTGAGCAAAGACCCCAACGAGAAGCGCGATCACATGGTCCTGCTGGAGTTCGTG
ACCGCCGCCGGGATCACTCTCGGCATGGACGAGCTGTACAAGTGA

>TMVΔCP-AfpB (sequence that replaces nucleotides 5757 to 6176 in TMV; AP24 is in blue)
ATGTCCAACAACATGGGCAACCTCAGGTCCTCCTTCGTCTTCTTCCTCCTGGCCCTGGTGACCTACACTTATGCA
AGTAAATACGGAGGACAATGCAGTCTGAAACACAACACGTGCACGTACCTGAAGGGTGGAAGAAACGTTATTGTC
AACTGCGGTTCGGCTGCCAATAAGAGGTGCAAGTCTGATCGCCACCACTGTGAATACGATGAGCACCACAGGAGG
GTTGACTGCCAGACTCCAGTTTGA

>TMVΔCP-AfpBKDEL (sequence that replaces nucleotides 5757 to 6176 in TMV; AP24 is in blue; KDEL is in green)
ATGTCCAACAACATGGGCAACCTCAGGTCCTCCTTCGTCTTCTTCCTCCTGGCCCTGGTGACCTACACTTATGCA
AGTAAATACGGAGGACAATGCAGTCTGAAACACAACACGTGCACGTACCTGAAGGGTGGAAGAAACGTTATTGTC
AACTGCGGTTCGGCTGCCAATAAGAGGTGCAAGTCTGATCGCCACCACTGTGAATACGATGAGCACCACAGGAGG
GTTGACTGCCAGACTCCAGTT**AAGGACGAACTC**TGA

>TMVΔCP-UP (sequence that replaces nucleotides 5757 to 6176 in TMV; AP24 is in blue)

ATGTCCAACAACATGGGCAACCTCAGGTCCTCCTTCGTCTTCTTCCTCCTGGCCCTGGTGACCTACACTTATGCA
GTTGACAGCCCTCAGTGGACCATCCAGAACGCCCAGCGTGTCTGCAACCCCCAAGACACCTCCTGCACCTGGACC
TTCAGCATCTACCCTGGCGCCGGTGCCGCCACTCCCTGCACCTACGTGGTTGAAGGCAGCCCTGCCTCCCGCGCC
AACGGTGGCCCCGTCACCTGCGGCGCATACACCGTCACTTCTGGCTGGAGCGGCCAGTTCGGTGCGGACAATGGC
TTCACCACTCTCTCTGTTGTCGACAACAACTCTCGCCAGATCATCTGGCCTGCTTATACCGATAAGCAGCTCGCT
GGCGGTGCGGTTGTCAAGCCTGACCAGAGCTACGCCCCTGCTGCTCTCCCTTGA

>TMVΔCP-UPKDEL (sequence that replaces nucleotides 5757 to 6176 in TMV; AP24 is in blue; KDEL is in green)
ATGTCCAACAACATGGGCAACCTCAGGTCCTCCTTCGTCTTCTTCCTCCTGGCCCTGGTGACCTACACTTATGCA
GTTGACAGCCCTCAGTGGACCATCCAGAACGCCCAGCGTGTCTGCAACCCCCAAGACACCTCCTGCACCTGGACC
TTCAGCATCTACCCTGGCGCCGGTGCCGCCACTCCCTGCACCTACGTGGTTGAAGGCAGCCCTGCCTCCCGCGCC
AACGGTGGCCCCGTCACCTGCGGCGCATACACCGTCACTTCTGGCTGGAGCGGCCAGTTCGGTGCGGACAATGGC
TTCACCACTCTCTCTGTTGTCGACAACAACTCTCGCCAGATCATCTGGCCTGCTTATACCGATAAGCAGCTCGCT
GGCGGTGCGGTTGTCAAGCCTGACCAGAGCTACGCCCCTGCTGCTCTCCCT**AAGGACGAACTC**TGA

>TMVΔCP-UPVS (sequence that replaces nucleotides 5757 to 6176 in TMV; AP24 is in blue; VS is in red)
ATGTCCAACAACATGGGCAACCTCAGGTCCTCCTTCGTCTTCTTCCTCCTGGCCCTGGTGACCTACACTTATGCA
GTTGACAGCCCTCAGTGGACCATCCAGAACGCCCAGCGTGTCTGCAACCCCCAAGACACCTCCTGCACCTGGACC
TTCAGCATCTACCCTGGCGCCGGTGCCGCCACTCCCTGCACCTACGTGGTTGAAGGCAGCCCTGCCTCCCGCGCC
AACGGTGGCCCCGTCACCTGCGGCGCATACACCGTCACTTCTGGCTGGAGCGGCCAGTTCGGTGCGGACAATGGC
TTCACCACTCTCTCTGTTGTCGACAACAACTCTCGCCAGATCATCTGGCCTGCTTATACCGATAAGCAGCTCGCT
GGCGGTGCGGTTGTCAAGCCTGACCAGAGCTACGCCCCTGCTGCTCTCCCT**GGCAACGGCCTCCTGGTCGACACC**
ATGTGA

>TMVΔCP-AgAFP (sequence that replaces nucleotides 5757 to 6176 in TMV; AP24 is in blue)
ATGTCCAACAACATGGGCAACCTCAGGTCCTCCTTCGTCTTCTTCCTCCTGGCCCTGGTGACCTACACTTATGCA
GCCACATACAATGGCAAATGCTACAAGAAGGATAATATCTGCAAGTACAAGGCACAGAGCGGCAAGACTGCCATT
TGCAAGTGCTATGTCAAAAAGTGCCCCCGCGACGGCGCGAAATGCGAGTTTGACAGCTACAAGGGGAAGTGCTAC
TGCTGA

>TMV(ToCP)-fGFP
(sequence that replaces nucleotides 5757 to 6176 in TMV; FLAG epitope in purple; linker in gray)
ATG**GACTACAAGGACGACGATGACAAA**GCAGCCGCTATGGTGAGCAAGGGCGAGGAGCTGTTCACCGGGGTGGTG
CCCATCCTGGTCGAGCTGGACGGCGACGTAAACGGCCACAAGTTCAGCGTGTCCGGCGAGGGCGAGGGCGATGCC
ACCTACGGCAAGCTGACCCTGAAGTTCATCTGCACCACCGGCAAGCTGCCCGTGCCCTGGCCCACCCTCGTGACC
ACCCTGACCTACGGCGTGCAGTGCTTCAGCCGCTACCCCGACCACATGAAGCAGCACGACTTCTTCAAGTCCGCC
ATGCCCGAAGGCTACGTCCAGGAGCGCACCATCTTCTTCAAGGACGACGGCAACTACAAGACCCGCGCCGAGGTG
AAGTTCGAGGGCGACACCCTGGTGAACCGCATCGAGCTGAAGGGCATCGACTTCAAGGAGGACGGCAACATCCTG
GGGCACAAGCTGGAGTACAACTACAACAGCCACAACGTCTATATCATGGCCGACAAGCAGAAGAACGGCATCAAG
GTGAACTTCAAGATCCGCCACAACATCGAGGACGGCAGCGTGCAGCTCGCCGACCACTACCAGCAGAACACCCCC
ATCGGCGACGGCCCCGTGCTGCTGCCCGACAACCACTACCTGAGCACCCAGTCCGCCCTGAGCAAAGACCCCAAC
GAGAAGCGCGATCACATGGTCCTGCTGGAGTTCGTGACCGCCGCCGGGATCACTCTCGGCATGGACGAGCTGTAC
AAGTGA
(ToMV sequence that replaces nucleotides 6366 to 6395 in TMV)
GAACCAAATCCTCAAAAAGAGGTCCGAAAAATAATAATAATTTAGGTAAGGGGCGTTCAGGCGGAAGGCCTAAAC
CAAAAAGTTTTGATGAAGTTGAAAAAGAGTTTGATAATTTGATTGAAGATGAAGCCGAGACGTCGGTCGCGGATT
CTGATTCGTATTAAATATGTCTTACTCAATCACTTCTCCATCGCAATTTGTGTTTTTGTCATCTGTATGGGCTGA
CCCTATAGAATTGTTAAACGTTTGTACAAATTCGTTAGGTAACCAGTTTCAAACACAGCAAGCAAGAACTACTGT
TCAACAGCAGTTCAGCGAGGTGTGGAAACCTTTCCCTCAGAGCACCGTCAGATTTCCTGGCGATGTTTATAAGGT
GTACAGGTACAATGCAGTTTTAGATCCTCTAATTACTGCGTTGCTGGGGTCTTTCGATACTAGGAATAGAATAAT
CGAAGTAGAAAACCAGCAGAATCCGACAACAGCTGAAACGTTAGATGCTACCCGCAGGGTAGACGACGCTACGGT
TGCAATTCGGTCTGCTATAAATAATTTAGTTAATGAACTAGTAAGAGGTACTGGACTGTACAATCAAAATACTTT
TGAAAGTATGTCTGGGTTGGTCTGGACCTCTGCACCTGCATCTTAAATGCATAGGTGCTGAAATATAAAGTTTGT
GTTTCTAAAACACACGTGGTACGTACGATAACGTATAGTGTTTTTCCCTCCACTTAAATCGAAAGGGTAGTGTCT
TGGAGCGCGCGGAGTAAACATATATGGTTCATATATGTCCGTAGCACGTAAAAAAGCGAGGGATTCGAATTCCCC
CGGAACCCCCGGGTTGGGGCCCA

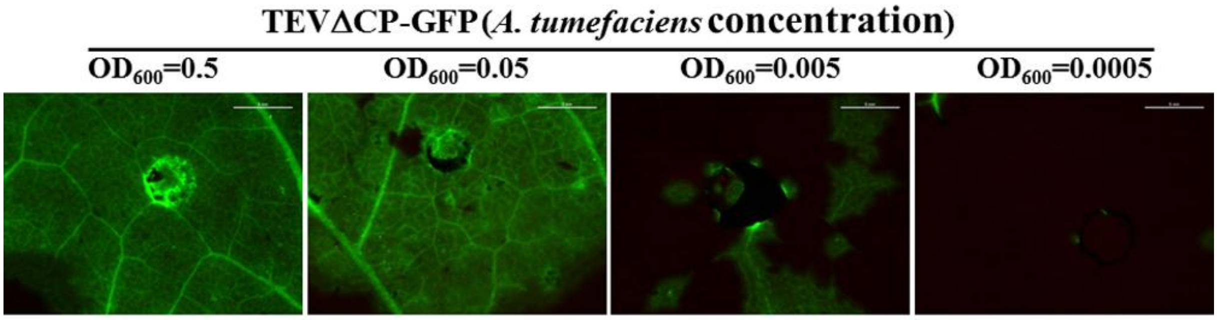

Figure S5. Dilution analysis of TMVΔCP-GFP infectivity in *N. benthamiana*. *A. tumefaciens* cultures to express TMVΔCP-GFP at the indicated OD_{600} were used to infiltrate *N. benthamiana* leaves. GFP fluorescence in the agroinfiltrated areas was detected at 7 dpi using a stereomicroscope. Representative pictures of three biological replicates are shown. Bars represent 5 mm.

Table S1. Primers used in this work.

Primer name	Sequence
**PI**	5’-TTACAAACGTGAGAGACGGAGGGCC-3’
**PII**	5’-GGAGGGAAAAACACTATGCGTTATC-3’
**PIII**	5’-GTAGAGAGAGACTGGTGATTTCAGC-3’
**CP:AP24_fwd**	5’-CTCAGTTCGTGTTCTTGTCAATGTCCAACAACATGGGC-3’
**AP24AfpB_rev**	5’ -TCCTCCGTATTTACTTGCATAAGTGTAGGT-3’
**AP24AfpB_fwd**	5’ -CACTTATGCAAGTAAATACGGAGGACAATGC-3’
**AfpB:CP_rev**	5’-CTACCTCAAGTTGCAGGACCTCAAACTGGAGTCTGGCAG-3’
**AfpBKDEL_rev**	5’-CCCGGGTTACTAGAGTTCGTCCTTAACTGGAGTCTGGCAGTC-3’
**KDEL:CP_rev**	5’-CTACCTCAAGTTGCAGGACCTCAGAGTTCGTCCTTAACTG-3’
**AfpD:CP_rev**	5’-CTACCTCAAGTTGCAGGACCTCAAGGGAGAGCAGCAGG-3’
**AfpDKDEL_rev**	5’-CCCGGGTTACTAGAGTTCGTCCTTAGGGAGAGCAGCAGGGGC-3’
**AfpDVS_rev**	5’-TCACATGGTGTCGACCAGGAGGCCGTTGCCAGGGAGAGCAGCAGGGGC- 3’
**VS:CP_rev**	5’-CTACCTCAAGTTGCAGGACCTCACATGGTGTCGACCAG-3’
**AgAFP:CP_rv**	5’-CTACCTCAAGTTGCAGGACCTCAGCAGTAGCACTTCCC-3’
